# Beyond the Edge: Basal‐Plane Defects as the Dominant Catalytic Sites in Sulfur‐Doped Graphene

**DOI:** 10.1002/advs.202521758

**Published:** 2026-03-02

**Authors:** Xuanhao Yuan, Chenhui Wang, Hui Hu, Hao Cui, Yan Li, Chengxin Wang

**Affiliations:** ^1^ State Key Laboratory of Optoelectronic Materials and Technologies School of Materials Science and Engineering Sun Yat‐sen University Guangzhou P. R. China

**Keywords:** defects in basal plane, density functional theory calculations, electrochemical reactions, inevitable oxygen‐bearing defects, sulfur‐doped graphene

## Abstract

Sulfur‐doped graphene (SG) has attracted considerable interest for energy conversion and storage applications. However, the relevant catalytic mechanisms remain obscure due to ongoing contentious debates regarding the location of dopant atoms. While theoretical studies often assume sulfur dopants preferentially reside at edge sites, experimental evidence consistently shows their homogeneous distribution throughout the carbon lattice. Here, we first demonstrated the thermal and dynamical instabilities of previously proposed models of S‐bearing defects in the basal plane of SG, which were used to explain experimentally observed enhanced lithium adsorption and magnetism. We then presented new stable defect configurations in the graphene lattice that incorporate both sulfur dopants and inevitable oxygen‐bearing functional groups, thereby explaining those experimental observations. These in‐plane defect models provide an internally consistent explanation for the active sites and catalytic mechanisms of oxygen, nitrogen, and sulfur reduction reactions, and suggest that the catalytic performance of SG cannot be rationalized solely by edge‐located sulfur dopants. In particular, several of the newly identified in‐plane defects exhibit calculated activities comparable to, or in some cases exceeding, those of representative edge configurations. Our findings highlight a previously underappreciated role of basal‐plane defects in sulfur‐doped carbonaceous materials, encompassing both metal‐free catalysts and graphene‐based single‐atom systems.

## Graphical abstract

Identification of basal‐plane sites in sulfur‐doped graphene challenges the conventional edge‐focused catalytic picture. Sulfur dopants together with inevitable oxygen‐containing groups modulate local charge and spin distributions, enhancing lithium binding and activating ORR/NRR intermediates. These in‐plane motifs also anchor long‐chain lithium polysulfides, providing unified design principles for chalcogen‐engineered graphene electrodes.

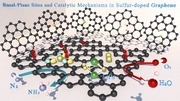

## 1 Introduction

The development of clean energy technologies—such as fuel cells and metal‐air batteries—is hindered by inefficient electrochemical reactions under conventional conditions, which currently rely on costly noble‐metal catalysts (e.g., Pt) [1, 2, 3]. To overcome limitations of scarcity, expense, and durability, earth‐abundant carbon‐based catalysts have emerged as promising alternatives [4, 5, 6]. While pristine graphene is electrochemically inert, heteroatom doping (e.g., N, S, B, O, and P) can activate its catalytic properties, enabling the design of carbon‐based metal‐free catalysts (CMFCs) [7, 8, 9, 10, 11, 12, 13, 14, 15, 16]. Beyond nitrogen, sulfur ranks as the second most extensively investigated dopant for tailoring the properties of carbonaceous materials [4, 9, 10, 11, 12, 13, 16, 17, 18, 19]. However, the specific location and precise role of sulfur heteroatoms within the carbon lattice remain debated.

On the one hand, there have been significant research efforts devoted to exploring the impact of S‐bearing defects in the basal plane of graphene on the physical and chemical properties of the host materials. For example, Tuček et al. found that introducing in‐plane S‐bearing defects into reduced graphene oxide (rGO) generated magnetism at low temperatures with S dopants occupying single‐carbon vacancies (SC_3_) [17]. In another work, Wang et al. reported that the same type of SC_3_‐type defect with substitutional S doping into the basal plane of graphene enhanced the lithium adsorption capability of graphene, thereby mitigating the issue of uneven lithium deposition [18]. Moreover, sulfur doping is also considered a powerful strategy to enhance the catalytic performance of M‐N‐C‐based single‐atom catalysts (SACs) [20, 21, 22, 23, 24, 25, 26]. The sulfur atoms, typically bonded with three neighboring atoms, are often believed to reside in the first or second coordination sphere around the metal centers. Nevertheless, those previously reported S‐bearing defects in the basal plane of graphene have not yet been demonstrated to be electrochemically active.

On the other hand, for over a decade, a prevailing consensus holds that the zigzag edge of graphene is a prerequisite for analyzing the catalytic performance of S‐doped carbonaceous materials toward the reduction of O_2_, N_2_, and S_8_ [19, 27, 28, 29, 30]. This view stemmed from the minimal electronegativity difference between carbon (C, 2.55) and sulfur (S, 2.58), suggesting little potential for significant enhancement of the catalytic performance of carbon atoms adjacent to basal plane S dopants [31]. However, this model suffers from two key shortcomings. While edge carbon atoms are demonstrably active for electrochemical reactions such as the oxygen reduction reaction (ORR) [32], their demonstrated activity calls into question the specific contribution of S doping to the observed activity. Moreover, the proportion of such edge carbon atoms is inherently limited in pristine graphene, which hinders its rational design for realistic applications [33]. Therefore, to enhance the electrochemical performance of S‐doped graphene (SG), the key lies in generating active sites within its basal plane. However, attempts to directly apply models of the SC_3_ configuration developed in the aforementioned fields—such as generating magnetism and suppressing lithium dendrite growth—to improve electrochemical performance are infeasible due to the inherently inert catalytic performance of this specific defect; thus, other structural features of the SG's basal plane also warrant consideration.

Numerous carbon precursors inherently contain oxygen within their molecular framework, causing such oxygen‐bearing functional groups to persist in the carbon matrix and enhance the catalytic performance [34, 35]. This phenomenon is particularly pronounced in thermally rGO [14, 36, 37, 38]. Compared to carbon and nitrogen, sulfur indeed exhibits a significantly larger covalent radius (S: 1.05 Å), whereas oxygen has a comparable atomic size to carbon and nitrogen (O: 0.66 Å; C: 0.76 Å; N: 0.71 Å) but higher electronegativity [39]. Consequently, in SG, both S dopants and inevitable residual O atoms (primarily from epoxy groups on the carbon surface) preferentially incorporate into carbon vacancies, resulting in more substantial distortion of the *sp* ^2^‐hybridized carbon framework than observed in nitrogen‐doped counterparts. This distortion creates electron‐deficient sites that facilitate trapping of additional oxygen groups, potentially forming active centers [40, 41]. Critically, whether these S/O‐coordinated defects can drive multi‐electron electrocatalytic processes, such as ORR and nitrogen reduction reaction (NRR) and sulfur redox reactions, remains unexplored, which provides a new opportunity for the design of novel in‐plane active sites in graphene.

By reproducing theoretical calculations from two representative studies [17, 18], we identified their proposed models for characterizing the structural feature of SG. However, our further calculations and analysis revealed that these models exhibited both thermodynamic and kinetic instability. Accordingly, based on experimentally confirmed structural features together with characteristic configurations of oxygen‐containing functional groups on carbon material surfaces, this study proposed a series of models of S/O‐bearing defects within the basal plane of SG and provided a systematic explanation for the observed experimental phenomena from the literature. After establishing the validity of our proposed defect structures, we revisited the catalytic mechanisms of SG in crucial electrochemical applications, including ORR in fuel cells, NRR in ammonia production and sulfur redox reactions in lithium–sulfur batteries, showing that basal‐plane defects can account for the enhanced activity without invoking specific edge motifs and may represent an important complementary class of active sites alongside edges in realistic materials. This work deepens our understanding of the origin of the enhanced catalytic performance of heteroatom‐doped carbons and accelerates their commercial applications.

## 2 Results and Discussion

### 2.1 Ineffective Model of Defect Configurations in SG

**FIGURE 1:**
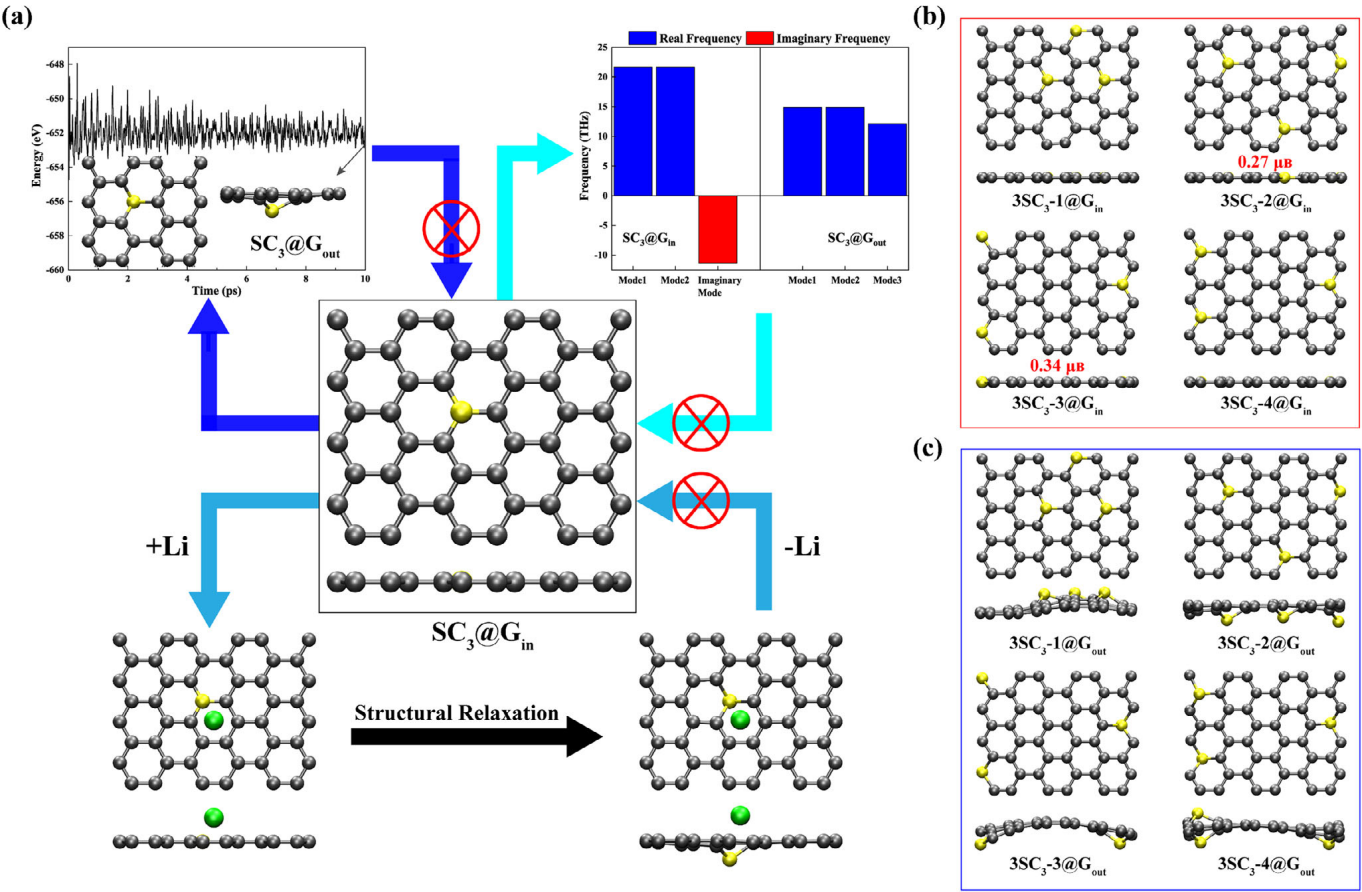
(a) Stability evaluation of the in‐plane substitutional S defect, SC_3_@G_in_. Center: relaxed SC_3_@G_in_ from Ref [18]. Top‐left: total‐energy profile from a 10 ps ab‐initio MD simulation at 300 K; the snapshot shows the S atom drifting out of the graphene plane (SC_3_@G_out_), which cannot be optimized to SC_3_@G_in_ (denoted by red cross). Top‐right: harmonic‐phonon analysis of the S atom in SC_3_@G_in_. SC_3_@G_in_ possesses one imaginary S‐localized mode (−11.3 THz; red) and two real modes (21.7 THz; blue), whereas SC_3_@G_out_ exhibits three real‐frequency modes (14.9, 14.9, and 12.1 THz) and no imaginary‐frequency modes. Bottom: Li adsorption drives the S atom of SC_3_@G_in_ out of plane; after Li removal, the relaxed structure of the substrate remains in the SC_3_@G_out_ state (hysteresis), which cannot be optimized to SC_3_@G_in_ (denoted by red cross). (b) Relaxed in‐plane 3SC_3_@G_in_ models (types 1–4) [Ref [17].]; top and side views; 3SC_3_‐2,3@G_in_ possess magnetism. (c) Corresponding out‐of‐plane 3SC_3_@G_out_ models (types 1–4). Color code: C (gray), S (yellow), Li (green).

Recently, in‐plane sulfur doping in graphene has been realized in two representative studies with different models. The first study by Wang et al. in 2019 reported SG suppressed lithium dendrite formation in lithium metal batteries [18], attributing this to strong lithium‐SG interactions enabling uniform deposition. However, we noted their model likely assumed sulfur substituted a carbon atom without breaking graphene's symmetry. This configuration, which can be denoted as SC_3_@G_in_ and is shown in Figure 1a, raises critical concerns: it not only lacks experimental validation but also contradicts the dynamic and thermodynamic instability revealed by our calculations. Detailed evidence supporting this contradiction will be presented in the following sections.

Although the side view of the SG model was not presented in Ref. [18], we could preliminarily infer from the top‐view characteristics that it adopted the model shown in Figure 1a (center). The most critical feature of this configuration is the coplanar arrangement of the sulfur atom with carbon atoms (SC_3_@G_in_). Based on this model of SC_3_@G_in_, we calculated the Li adsorption energy (*E*_ads_ (Li)) to be −3.59 eV, which is consistent with that from the literature (−3.31–−3.46 eV for various sites around S dopant). It confirms that Ref. [18] employed the same model of SC_3_@G_in_ as shown in Figure 1a (center). Since *E*_ads_ (Li) is more negative than the cohesive energy of bcc Li (*E*_coh_ (Li) = −1.6 eV), Wang et al. believed that Li atoms preferred surface adsorption over bulk cohesion, benefiting the suppression of the lithium dendrite growth [18]. However, we found that such low *E*_ads_ (Li) originated not from strong Li adsorption in the SC_3_@G_in_ model, but from intrinsic flaws in its planar assumption, as reflected by the following calculations. First, ab initio MD simulations (AIMD) revealed sulfur protrudes > 1.7 Å from the carbon plane at 300 K, named as SC_3_@G_out_ (Figure 1, top‐left). Structural optimization failed to restore planarity, indicating a metastable defect model of SC_3_@G_in_. Second, phonon analysis showed one imaginary frequency of −11.3 THz for one of three sulfur vibrational modes (Figure 1a, top right), confirming dynamic instability. The eigenvector indicates out‐of‐plane displacement, lowering the energy and favoring SC_3_@G_out_ configuration. Third, Li adsorption triggered an irreversible transition (Figure 1a, bottom). Upon Li adsorption on SC_3_@G_in_, sulfur shifted out‐of‐plane. After Li desorption, the system stabilized irreversibly in the SC_3_@G_out_ configuration, demonstrating hysteresis behavior. More crucially, SC_3_@G_out_ is energetically favored by 2.04 eV, explaining the artificially low *E*_ads_ (Li) in planar models due to unphysical strain.

These results demonstrate that the artificially constrained SC_3_@G_in_ model fails to describe the ground state of SG. The thermodynamically stable SC_3_@G_out_ configuration (energy advantage: 2.04 eV) more accurately represents the reality. For SC_3_@G_out_, we recalculated *E*_ads_ (Li) to be −1.55 eV, which was less negative than *E*_coh_ (Li) = −1.6 eV. This finding reveals that the low adsorption energy of Li atom in SG from the literature [18] was caused by the deformation energy of unstable SC_3_@G_in_ instead of the strong binding strength. That is to say that SC_3_@G_in_ cannot explain the strong lithium binding observed experimentally. Consequently, defect configurations beyond that should be investigated to elucidate the atomic‐scale origin of lithium‐trapping capability of SG.

Further experimental evidence for sulfur incorporation into the graphene carbon lattice was reported by Tuček et al., who detected magnetism in sulfur‐doped reduced graphene oxide (SrGO), containing more oxygen‐bearing groups than graphene on the carbon lattice, and proposed four distinct defect models (3SC_3_‐*n*@G_in_, *n* = 1, 2, 3, and 4) with 3S dopants in the cell as shown in Figure 1b [17]. These models exhibited magnetic ground states and structural similarities to the configuration (Figure 1a), where S atoms substitute carbon atoms within the graphene plane. Consequently, these planar S‐doping models were used to investigate the origin of the experimentally observed magnetism. Our DFT calculations confirmed that S atoms in these four model configurations preferentially reside in the graphene plane. In addition, the 3SC_3_‐2@G_in_ and 3SC_3_‐3@G_in_ configurations exhibit finite net magnetic moments of 0.27 and 0.34 µ_B_ per supercell, respectively, with spin density localized on S and adjacent C atoms (see Figure S1). However, AIMD simulations and phonon analyses demonstrate dynamical and thermal instability (Figure S2). Therefore, we proposed that the thermodynamically stable configuration involved sulfur atoms protruding out of the graphene plane. Therefore, we established corresponding stable configuration of SG featured sulfur atoms protruding out‐of‐plane Figure 1c, with the 3SC_3_‐*n*@G_out_ (*n* = 1, 2, 3, and 4) structures exhibiting significant energy advantages over planar counterparts with Δ*E* = −7.58 eV (3SC_3_‐1@G_out_), −7.87 eV (3SC_3_‐2@G_out_), −8.37 eV (3SC_3_‐3@G_out_), and −7.65 eV (3SC_3_‐4@G_out_). More importantly, these thermodynamically stable configurations universally adopt non‐spin‐polarized ground states, thus invalidating prior magnetic mechanism proposals from the literature [17].

Generally, previously proposed SC_3_@G_in_ defect models, invoked to explain Li adsorption and magnetism in sulfur‐doped graphene, shared a common shortcoming: they lack validation through stability calculations. In contrast, the corresponding stable SC_3_@G_out_ structures exhibit weak interaction with Li atoms and possesses a non‐spin‐polarized ground state. Taken together, these findings suggest that the previously proposed model cannot be utilized to explain the experimental results, highlighting the need for more realistic defect models to rationalize the experimentally observed properties.

### 2.2 Stable Defect Configurations for Trapping Li Atoms and Magnetism in SG

**FIGURE 2:**
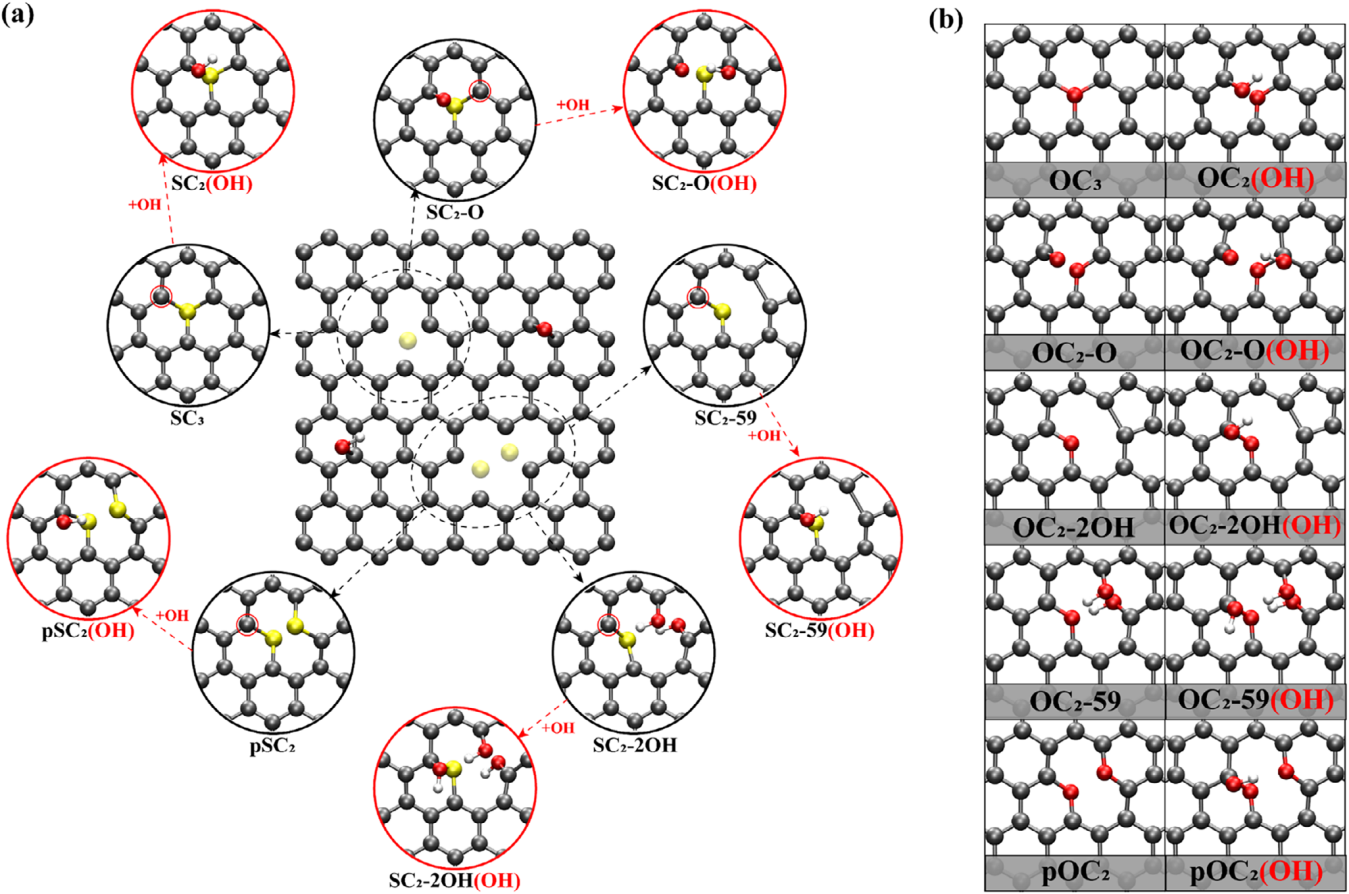
Possible defect configurations in SG proposed in the current study. (a) S‐bearing basal‐plane defects. Center: SVs and DVs in graphene for S dopants (center, dashed outline) and inevitable functional groups of epoxy and hydroxyl. Outer sphere: five possible S‐bearing configurations of SC_3_, SC_2_‐O, SC_2_‐59, SC_2_‐2OH, and pSC_2_ formed by depositing S into SVs and DVs (in black solid circles) and their hydroxylated derivatives [SC_2_(OH), SC_2_‐O(OH), SC_2_‐59(OH), SC_2_‐2OH(OH), and pSC_2_(OH)] (in larger red solid circles). Smaller red circles denote those *sp* ^3^‐like C atoms trapping ─OH. (b) O‐bearing basal‐plane defects. Analogous oxygen‐containing defects (OC_3_, OC_2_‐O, OC_2_‐59, OC_2_‐2OH, and pOC_2_) due to the O deposition in SVs and DVs (left column) and their hydroxylated derivatives (right column).

Although experimental work has demonstrated that introducing sulfur impurities enables graphene to suppress lithium dendrite growth [18] and exhibit magnetism [17], our series of calculations has shown that the model of SC_3_@G_in_ used in the literature is unstable, while the corresponding stable defect configuration of SC_3_@G_out_ cannot explain the experimental observations. This indicates that the current understanding of the structural characteristics of SG is insufficient. So, it is essential to explore other possible structural features beyond SC_3_@G_out_ in the graphene lattice. Actually, the incorporation of sulfur dopants into the carbon lattice primarily occurs through filling carbon vacancies. As shown in the center of Figure 2, we considered that the graphene or rGO may contain single vacancies (SVs) and double vacancies (DVs) for S dopants as well as unavoidable oxygen‐bearing functional groups of epoxy (─O─) and hydroxyl (─OH) [42]. When a single sulfur atom adsorbs on an SV, it may form an SC_3_ structure, whereas filling a double vacancy may result in SC_2_‐59 as well as SC_2_‐2OH with two carbon atoms saturated with OH as shown in black circles of Figure 2a. If two sulfur atoms occupy DVs, a pSC_2_ structure may form. In addition, we also proposed another possible defect of SC_2_‐O considering the presence of carbonyl group (C═O) [43, 44]. Since the sulfur atom has a larger atomic radius than carbon and tends to protrude from the graphene plane, the carbon atoms adjacent to S dopants exhibit *sp* ^3^‐like bonding characteristics, leaving a dangling bond that needs to be saturated. Given the inevitable hydroxyl on the carbon lattice of graphene, those *sp* ^3^‐like C atoms (in smaller red circles of Figure 2a) in S‐bearing defects of SC_3_, SC_2_‐O, SC_2_‐59, SC_2_‐2OH and pSC_2_ tend to adsorb unavoidable OH on graphene at the C atoms adjacent to S dopant, leading to SC_2_(OH), SC_2_‐O(OH), SC_2_‐59(OH) and SC_2_‐2OH(OH) and pSC_2_(OH) (in larger red circles of Figure 2a). The corresponding adsorption energies of OH range from −1.55 to −2.53 eV lower than that on pristine graphene (−1.23 eV), indicating significantly stronger trapping capability for OH (see Table S1). Additionally, sulfur doping induced carbon vacancies may also be filled by inevitable oxygen atoms adsorbed on the surface of graphene. Given the similar chemical properties of oxygen and sulfur, sulfur‐doped graphene may also contain analogous oxygen‐bearing defects, with formation mechanisms similar to those of sulfur‐bearing defects [40], as shown in Figure 2b. The presence of such O‐bearing defects can be corroborated by the structural features of ‐C‐O‐C‐ and C = O found by previous experimental results about S‐doped graphene/rGO [36]. Our DFT calculations further demonstrated that the presence of most defect configurations shown in Figure 2 is feasible to be fabricated, as confirmed by the cohesive energies (see Figure S3), which are comparable or lower than that of SC_3_.

**FIGURE 3:**
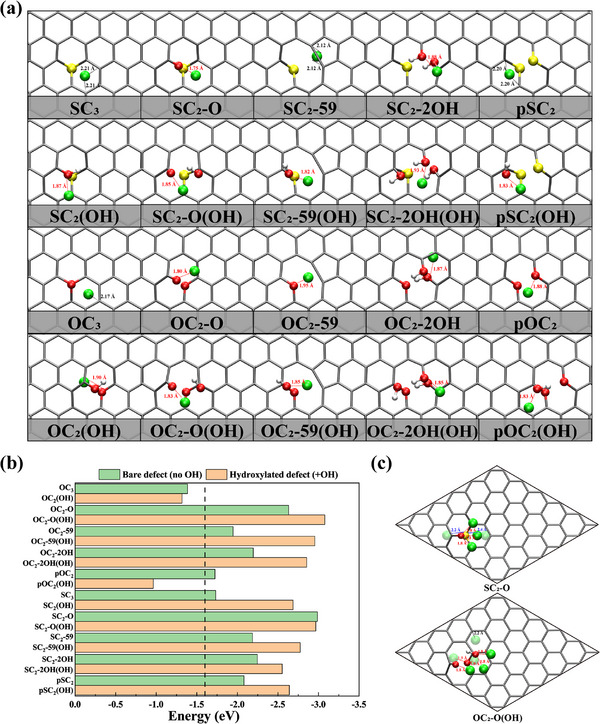
(a) Top views of Li adsorption on S‐ and O‐bearing defects in SG. The current position corresponds to an independent local minimum obtained from a site scan followed by full relaxation. (b) Minimum Li‐adsorption energies *E*_ads_ (Li) for each defect. The vertical dashed line at −1.60 eV marks the cohesive energy of bcc Li; bars extending to the right, more negative than −1.60 eV indicate stronger binding than Li metal. (c) Competitive adsorption sites of Li in the extended region of SC_2_‐O (top) and OC_2_‐O(OH) (bottom). The annotated bond lengths denote the shortest distances between Li and its nearest neighboring atoms (O, S, C), highlighted with dashed lines: Li–O in red, Li–S in blue, and Li–C in black.

We next reassessed SG's ability to suppress lithium dendrites via the Li‐adsorption energies of all defects depicted in Figure 2. For each defect, multiple adsorption sites were probed with the adsorption energies summarized in Table S2, whereas Figure 3a displayed only the most stable site. DFT calculations revealed that the *E*_ads_(Li) (from −1.7 to −3.1 eV) for most S‐bearing and O‐bearing defects are significantly lower than the cohesive energy of bcc Li (−1.60 eV) (see Figure 3b), indicating that the abundant defect structures within the carbon lattice of SG markedly enhance the suppression of lithium dendrites. As Figure 3b shows, hydroxylation markedly boosts Li binding for almost every defect, pushing *E*_ads_ far below the bcc Li threshold. Figure 3c illustrates the trapping capabilities of two representative defects for Li atom with the lowest adsorption energies: the O‐based OC_2_‐O(OH) (*E*_ads_ (Li) = −3.08 eV) and the S‐based SC_2_‐O (*E*_ads_ (Li) = −2.99 eV). For each defect, we showed several possible adsorption sites of Li with *E*_ads_ (Li) < −1.60 eV, with the shortest Li–O/S/C distances annotated. In the most stable geometry of both defects, Li is stabilized by a 1.8 Å Li‐O contact—toward the hydroxyl oxygen in OC_2_‐O(OH) and the lattice oxygen in SC_2_‐O. In Figure 3c, dashed lines color‐code the nearest neighbor: red for Li–O, blue for Li‐S, and black for Li–C. Moreover, other defects such as pSC_2_(OH), pOC_2_(OH), SC_2_‐O, and SC_2_‐59 also provide extended zones of strong Li binding (Figure S4), promoting uniform Li deposition and further mitigating dendrite formation. These results demonstrate that SG possesses comparable or better performance compared to other heteroatom‐doped graphene considering the ability to suppress Li dendrites. For example, *E*_ads_(Li) for N and/or B‐doped graphene with carbon vacancy spans a wide range of −0.99–−3.12 eV, and N‐doped graphene even shows unfavorable Li adsorption (0.75 eV) [45, 46].

These results indicate that the suppression of lithium dendrite formation in SG is primarily attributable to the defect configurations shown in Figure 2, rather than to the SC_3_ model previously proposed in the literature [18]. This mechanism can also be extended to explain the enhanced electrochemical performance of sulfur‐doped carbonaceous materials in sodium and potassium ion batteries [47, 48], although it is out of the scope of the current study.

**FIGURE 4:**
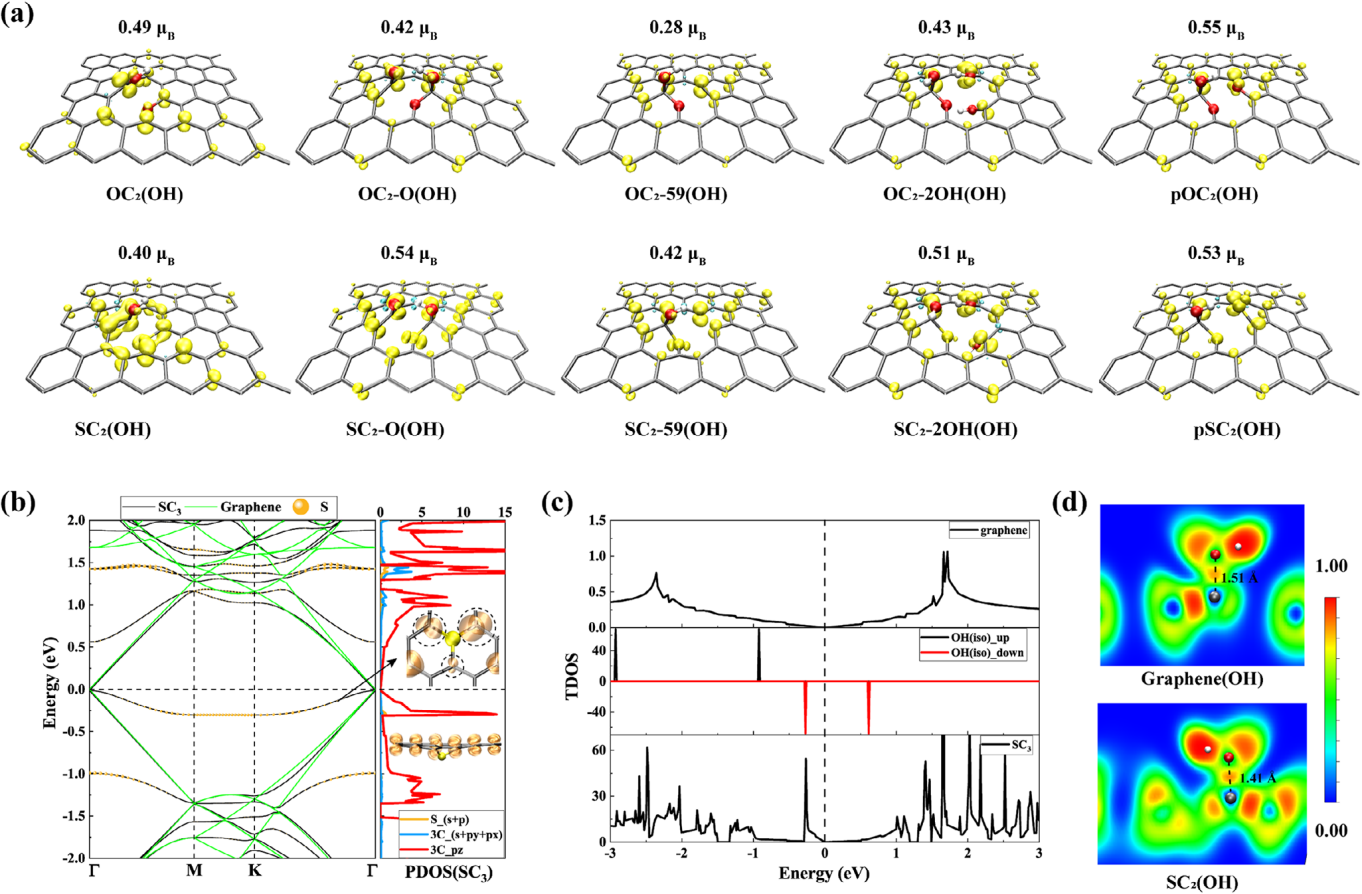
(a) Spin‐density isosurfaces for defects in the basal plane of SG. Yellow lobes indicate regions of largest spin polarization near the heteroatom and neighboring C sites; numbers above each panel are the total magnetic moments (*µ*_B_) per supercell. (b) Band structures along Γ‐M‐K‐Γ for pristine graphene (green) and the SC_3_ defect (black). Right: PDOS projected onto S and its three neighboring C atoms (C_*pz* and C_(*s*+*px*+*py*)), showing a flat band at ∼ −0.3 eV relative to the Fermi level (dashed line). Inset: partial charge density of this defect‐derived state on S and the adjacent C atoms. From the side view, it is evident that the introduction of the S defect induces an out‐of‐plane charge protrusion on the neighboring C atoms. (c) Total DOS (TDOS) of pristine graphene (top), spin‐resolved TDOS of isolated OH (middle), and SC_3_ (bottom). (d) ELF maps for OH adsorption on pristine graphene (top) and SC_2_(OH) (bottom). Compared with graphene (C‐O = 1.51 Å), the C─O bond in SC_2_(OH) shortens to 1.41 Å and shows enhanced electron localization (red region), confirming stronger chemical bonding at the defect site.

Regarding another representative study on the magnetism of SrGO, it was initially attributed to the S‐bearing defects of 3SC_3_@G_in_ in the basal plane of SrGO, which however were proved to be dynamically unstable in the previous section (see as shown in Figure 1b,c). Moreover, the corresponding stable configurations of 3SC_3_@G_out_ are not magnetic, and thus the experimentally observed magnetism in SG must be attributed to other structural features of SrGO. It has been reported that adsorption of OH on the basal plane of graphene lattice can disrupt the π‐bond balance and yield magnetic moments [49, 50]. Therefore, it is reasonable to examine the magnetism of those OH‐regulated defects proposed in the current study, as shown in Figure 2. Our DFT calculations demonstrated that XC_2_(OH), XC_2_‐O(OH), XC_2_‐59(OH), XC_2_‐2OH(OH), and pXC_2_(OH) (X = S, O) possessed spin‐polarized ground state with a total magnetism of 0.28–0.55 µ_B_ (see Figure 4a). Moreover, the spin density is mainly distributed around S/O defects in the graphene lattice, with the C atoms connected to the OH group possessing the most magnetism.

Overall, we believe that incorporating S dopants into the graphene basal plane strengthens the OH adsorption, thereby disrupting the π‐bond balance and yielding magnetic moments. This mechanism provides a plausible explanation for the experimentally observed magnetism in SrGO [17]. As shown in Figure 4b, the SC_3_ defect introduces a defect‐derived flat band at ∼−0.3 eV relative to the Fermi level. This state is dominated by the C_*p_z_* orbital contribution, with additional hybridization from S_(*s* + *p*) and three neighboring C_(*s* + *px* + *py*) orbitals. The inset partial charge density confirms that S‐doping induced defect state gives rise to more localized charge around C atoms adjacent to S, which shows dumbbell‐shaped and extends further into space than the rest C atoms. This indicates that the C atoms adjacent to the S dopant tend to form a stronger bond with OH than the rest C atoms. Moreover, as shown in Figure 4c, the density of states (DOS) of SC_3_ exhibits pronounced peaks located much closer to the Fermi level than those in pristine graphene. This energetic proximity suggests that SC_3_ can hybridize more effectively with the frontier orbitals of OH, thereby strengthening the chemical bonding. This conclusion is also supported by the OH binding energies (Table S1; SC_3_: −2.37 eV versus graphene: −1.23 eV) and markedly enhanced electron localization between C and O evidenced by the Electron localization function (ELF) maps (see Figure 4d). The different binding strength can also be reflected by the C─O bond length, which is 1.51 Å in graphene (OH) and 1.41 Å in SC_2_(OH).

Critically, since SrGO was synthesized by thermally treating graphite oxide in nitrogen using H_2_S as the sulfur source [17], S‐bearing defects like SC_3_ can trap and anchor a greater number of OH groups than rGO, even after this thermal annealing process. Consequently, the magnetic contribution from OH adsorption in undoped rGO is comparatively low [17, 35]. These results clarify the microscopic link between sulfur doping, hydroxyl chemisorption, and magnetism, and suggest a practical route to tune magnetism in 2D carbons.

It should be noted that the experimentally observed magnetism induced by sulfur doping [17] is definitely not attributed to the intrinsic defects in graphene, such as zigzag edges or vacancy defects. This can be corroborated by a recent experimental study reported that intrinsic defects in graphene, including zigzag edges or vacancy defects, can induce magnetism, whereas sulfur impurities tend to eliminate such magnetism [51]. Integrating our results with literature findings, we revealed that sulfur doping can have dual effects on graphene magnetism—either generating or quenching it—depending on the atomic site of sulfur in the carbon lattice.

Overall, such defects in the basal plane of SG shown in Figure 2 demonstrate enhanced Li adsorption strength and magnetic characteristics, which not only explain the experimental results but also enable us to establish novel modeling principles for the defects from the basal plane of SG [17, 18]. Therefore, it is essential to further identify the basal‐plane sites and revisit the mechanism of electrochemical reactions catalyzed by SG reported in the literature.

### 2.3 Revisiting the Mechanism of Electrochemical Reactions of ORR/NRR Over SG

The superior catalytic performance of SG for various electrochemical reactions, including ORR [16, 19], sulfur reduction reaction (SRR) [52], and NRR [53], has been experimentally demonstrated. And the active sites were assumed to be located at the edges, particularly at zigzag edges. In this work, we explicitly distinguish edge sites from basal‐plane defects based on geometric topology. Here, edge sites refer to atoms located at the open boundaries of graphene sheets, such as zigzag or armchair edges, where the *sp* ^2^ lattice is terminated. In contrast, basal‐plane defects are defined as sulfur‐containing structures embedded within the continuous graphene lattice, where sulfur occupies intrinsic in‐plane carbon vacancies rather than terminating the sheet. However, this prevailing understanding of the edge‐hosted active sites confronts two challenges. First, zigzag edge defects constitute only a minor fraction of the whole SG, offering severely limited catalytic sites. Consequently, enhancing active site density necessitates shifting research emphasis to basal‐plane defects, which have not been investigated yet. Second, the origin of the enhanced catalytic performance of SG is still under debate [16, 19, 54]. On the one hand, the zigzag edge sites are inherently catalytically active due to the localized edge states, which exhibit stronger binding affinities toward small molecules and reaction intermediates compared to pristine *sp* ^2^‐hybridized carbon atoms in the basal plane [30]. On the other hand, substitutional sulfur atoms at such edges may decrease the amount of active sites, thereby diminishing, rather than enhancing, ORR performance [19, 30, 51]. This further indicates that the enhanced ORR performance of SG observed in the experiment might be attributed to the active sites from the basal plane of graphene [16]. Consequently, a systematic investigation of the catalytic performance of our newly found defects in SG (Figure 2) is imperative for a comprehensive understanding of the role of S doping.

**FIGURE 5:**
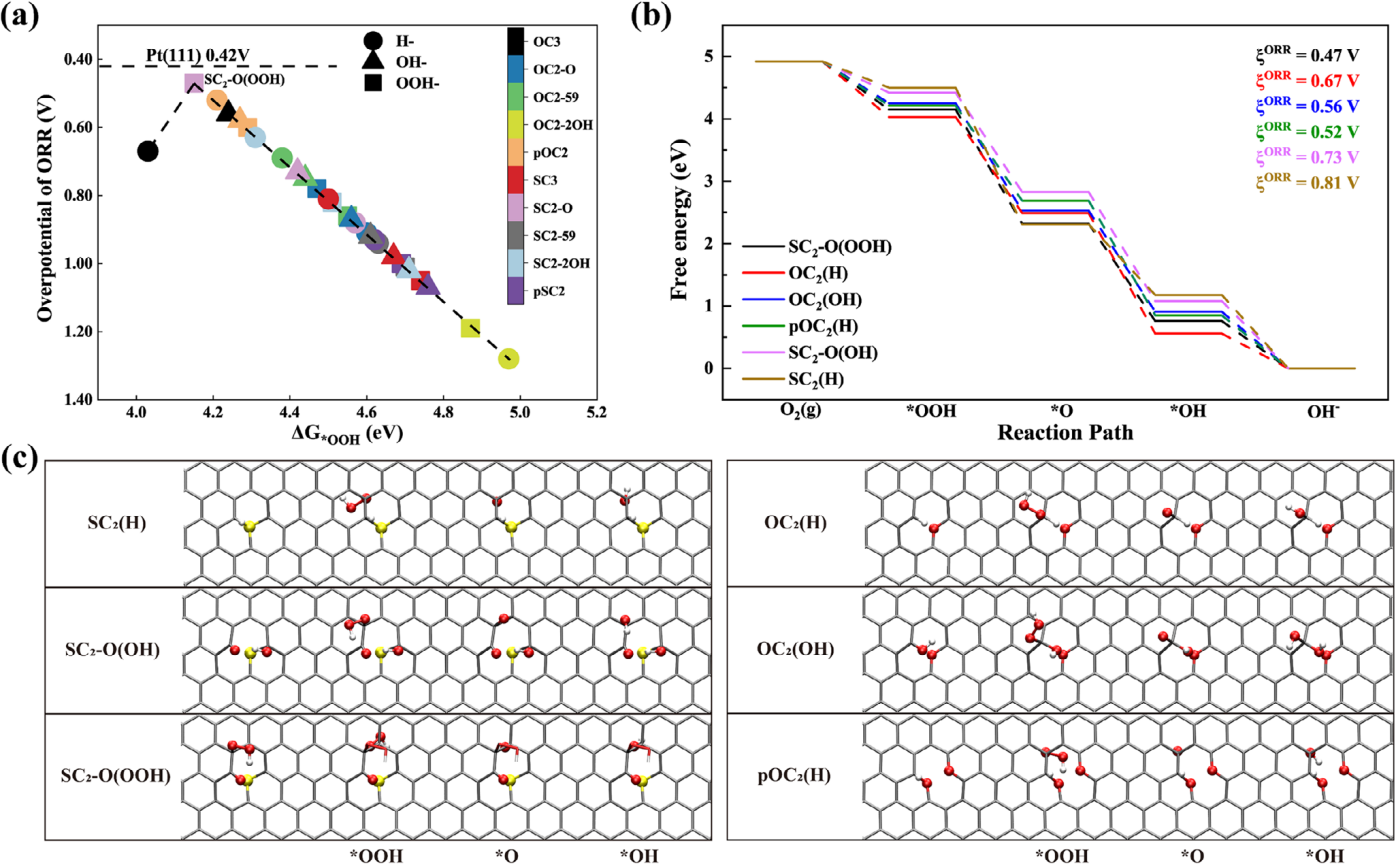
(a) Volcano plot of ORR overpotential (ξ^ORR^) versus Δ*G* ^*^OOH for all basal‐plane defects in SG. Circles, triangles, and squares represent H‐, OH‐, and OOH‐modified 10 typical defects in SG (XC_3_, XC_2_‐O, XC_2_‐59, XC_2_‐2OH, pXC_2_ (X = S, O)), respectively; point colors identify defect families (legend). The Pt (111) benchmark (0.42 V) is shown by the grey dashed line; the best ORR performance is from SC_2_‐O(OOH) with ξ^ORR^ = 0.49 V. (b) Free‐energy profiles at U = 0 V for six most active defects, with ξ^ORR^ values listed on the right. (c) Top‐view of adsorbed ORR reaction intermediates on six defects.

Charge and spin density redistribution are responsible for the ORR performance of CMFCs [19]. We have demonstrated that various S‐ and O‐bearing defects exhibit spin‐polarized ground states upon adsorption of ^*^OH. Inspired by these findings, we hypothesized that the adsorption of ^*^H (acidic condition) and ^*^OOH intermediates during the ORR process may also occur inducing spin polarization with more information available in our previous work [40]. Consequently, we examined 29 defects of OH‐, H‐, and OOH‐functionalized XC_3_, XC_2_‐O, XC_2_‐59, XC_2_‐2OH, pXC_2_ (X = S, O), which were expected to exhibit superior ORR performance due to the redistribution of spin density. We then performed systematic DFT calculations of the adsorption free energies of ORR intermediates on those defects, and more details about the structures and adsorption free energies are available in the supporting information (Figures S5–S14 and Table S3). Our DFT calculations showed that SG can effectively catalyze ORR as reflected by overpotentials *ξ* ^ORR^ < 1.23 V vs. reversible hydrogen electrode (RHE) for quite a few active sites. The overpotentials show a perfect volcano variation with the adsorption free energy of hydroperoxyl (ΔG_*OOH_), demonstrating that almost all ORR processes possess the potential determining step (PDS) of O_2_ → OOH^*^. As shown in Figure 5, the best ORR performance is from SC_2_‐O(OOH), giving rise to *ξ* ^ORR^ = 0.49 V, which is even comparable to that of 0.42 V for Pt (111) [40] and much better than metallic catalysts such as FeN_4_ (*ξ* ^ORR^ = 0.62 V) [55]. This predicted superior ORR performance of various defects in SG is consistent with the experimental results [16]. Moreover, AIMD simulations were carried out for SC_2_‐O(OOH) with ^*^OOH, ^*^O, and OH intermediates, confirming their thermal stability on this defect at 300 K (see Figure S15). While other defects also exhibit competitive catalytic performance, such as OC_2_(H) (*ξ* ^ORR^ = 0.67 V), OC_2_(OH) (*ξ* ^ORR^ = 0.56 V), pOC_2_(H) (*ξ* ^ORR^ = 0.52 V), SC_2_‐O(OH) (*ξ* ^ORR^ = 0.73 V), and SC_2_(H) (*ξ* ^ORR^ = 0.81 V). The overpotentials for the rest defects are listed in Table S3. The free energy changes of ORR over five selected defects of SC_2_‐O(OOH), OC_2_(H), OC_2_(OH), pOC_2_(H), SC_2_‑O(OH), and SC_2_(H) are shown in Figure 5b, and the adsorption configurations of reaction intermediates are presented in Figure 5c.

To address the edge‐versus‐basal‐plane ambiguity, we further benchmarked our basal‐plane defects against explicit graphene edge models. Using sulfur‐doped graphene nanoribbons with well‐defined zigzag and armchair terminations, we found that the ORR overpotentials for zigzag edges are 0.54 V (S on the same side of the reaction site) and 0.55 V (the opposite side without S), indicating only a marginal difference. In contrast, for the armchair edge, OOH^*^ cannot be stably adsorbed and relaxes to a desorbed state, exhibiting poor ORR performance. These results are inconsistent with the experimental results that S doping can enhance the ORR performance of graphene [16]. Therefore, we believe that high ORR activity of sulfur‐doped graphene can intrinsically originate from basal‐plane defects rather than physical edges (see Figure S16).

These findings also highlight the importance of O‐containing defects such as OC_2_(OH) and OC_2_‐O(OH) in promoting ORR activity in carbon‐based metal‐free catalysts (CMFCs) containing the feature of C═O and C─O [56, 57, 58]. Moreover, this mechanism can also be confirmed by earlier experimental observations where undoped rGO [12] and carbon cloth [35] exhibited significant ORR activity, likely due to the inevitable oxygen‐related defects during material synthesis. A very recent experimental work shows that inevitable O‐bearing functional group of carbonyl plays a crucial role in catalyzing ORR [38]. This confirms again the contribution of our newly identified defects tailored by O‐bearing functional groups such as hydroxyl and carbonyl.

The electrochemical NRR offers a sustainable route for ammonia production under ambient conditions, significantly reducing the energy demand compared to the conventional Haber–Bosch process [59, 60, 61]. As promising alternatives to metal catalysts, CMFCs have also been investigated for their applications in catalyzing NRR [62]. While early theoretical and experimental efforts primarily focused on B‐, N‐, or O‐doped graphene [63, 64, 65], studies on SG have only recently attracted attention [66]. However, most of these works adopt simplified edge‐centric models in which sulfur atoms are assumed to be doped into edges—disregarding experimental evidence showing that sulfur can be incorporated uniformly into the basal plane and is often accompanied by oxygen‐containing functional groups (e.g., C═O, ─OH). Such an edge‐only modeling approach hinders further understanding of the structure–activity relationship in SG.

**FIGURE 6:**
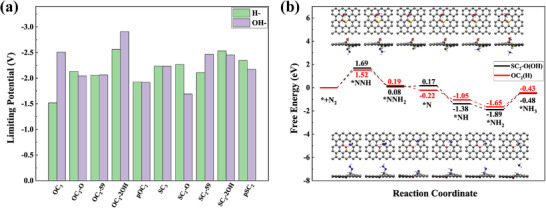
(a) Limiting potentials U_L_ of NRR over H‐ and OH‐ modified 10 typical defects in SG (XC_3_, XC_2_‐O, XC_2_‐59, XC_2_‐2OH, pXC_2_ (X = S, O)). (b) Free‐energy changes of NRR (U = 0 V) on two sites of OC_2_(H) (red) and SC_2_‐O(OH) (black). In both cases, the ^*^N_2_ → ^*^NNH is the rate‐limiting step, setting U_L_ at −1.52 and −1.69 V, respectively.

Inspired by aforementioned performance of SG for ORR, we then explored the NRR performance of 20 OH‐ and H‐functionalized XC_3_, XC_2_‐O, XC_2_‐59, XC_2_‐2OH, pXC_2_ (X = S, O) (see Figures S17–S26). Gibbs free‐energy profiles of NRR were computed along the distal pathway:N_2_→^*^NNH→^*^NNH_2_→^*^N→^*^NH→^*^NH_2_→^*^NH_3_, using the computational hydrogen electrode (CHE) framework (T = 298 K, pH 0). Note that other reaction pathways were not explored due to the missing of side‐on adsorption configuration of ^*^NNH. Our calculations showed that those defects in SG basal plane gave rise to the limiting potential ranging from −2.91 to −1.52 V (Figure 6a). Among them, OC_2_(H) and SC_2_‐O(OH) are identified as the most promising active sites, featuring limiting potentials of −1.52 and −1.69 V, respectively. The limiting potentials obtained in the current study are even close to the −1.53 V based on edge‐S models [53]. However, basal‐plane defects, unlike edge‐confined motifs, can be dispersed throughout the graphene sheet, offering a more broadly accessible and versatile catalytic platform.

Overall, the basal‐plane defects in SG have been identified as catalytically active for ORR and NRR. The construction of such defects (etc., SC_3_, SC_2_‐O and OC_2_‐O) is based on experimentally confirmed structural characteristics in SG, including ─C─S─C, C─O and C═O bonds [36]. These defects share a common feature of the incorporation of S/O heteroatoms into the carbon lattice. This induces effective adsorption of OH/H species on S/O‐adjacent carbon atoms, generating active sites with localized charge and/or magnetic moments (Figure 4a–c). Consequently, those sites readily adsorb polar intermediates and catalyze the reactions. This defect‐centric modeling strategy extends the traditional edge‐centric picture of active sites in SG by explicitly incorporating basal‐plane defects into the discussion. In other words, basal‐plane defects—beyond the previously hypothesized requirement for sulfur at graphene edges—represent an additional, non‐negligible family of potential ORR/NRR active sites in SG. Moreover, this modeling strategy for SG also accounts for the ORR activity observed in undoped carbonaceous materials in prior studies, as various inevitable oxygen‐containing defects are likewise active for ORR [12, 35].

### 2.4 Revisiting the Mechanism of SRR Catalyzed by SG

The practical application of lithium–sulfur (Li–S) batteries is hindered by the notorious shuttle effect, arising from the dissolution and diffusion of long‐chain lithium polysulfides (LiPSs) including Li_2_S_n_ (n = 4, 6, and 8) between the cathode and anode during cycling. Recent experimental studies have demonstrated that incorporating SG into the cathode significantly strengthens the binding affinity for LiPSs, thereby suppressing the shuttle effect [52, 67]. However, the underlying mechanisms governing these electrochemical reactions remain poorly understood, largely due to the oversimplified assumptions in current modeling strategies. Those prevailing theoretical models, as proposed in prior studies, predominantly assume that sulfur dopants and following adsorption of LiPSs are located at graphene edges [29, 67]. Such simplifications fail to explain the experimentally observed homogeneous distribution of sulfur dopants across the graphene basal plane. Moreover, oxygen‐containing functional groups (e.g., C═O, ─OH), ubiquitous in experimentally synthesized SG, are typically neglected in these models despite their potential cooperative role with sulfur in LiPSs adsorption. Most critically, such edge‐limited models cannot rationalize the shuttle‐suppression capability of SG due to weak interactions between edge sulfur dopants and long‐chain LiPSs [29].

**FIGURE 7:**
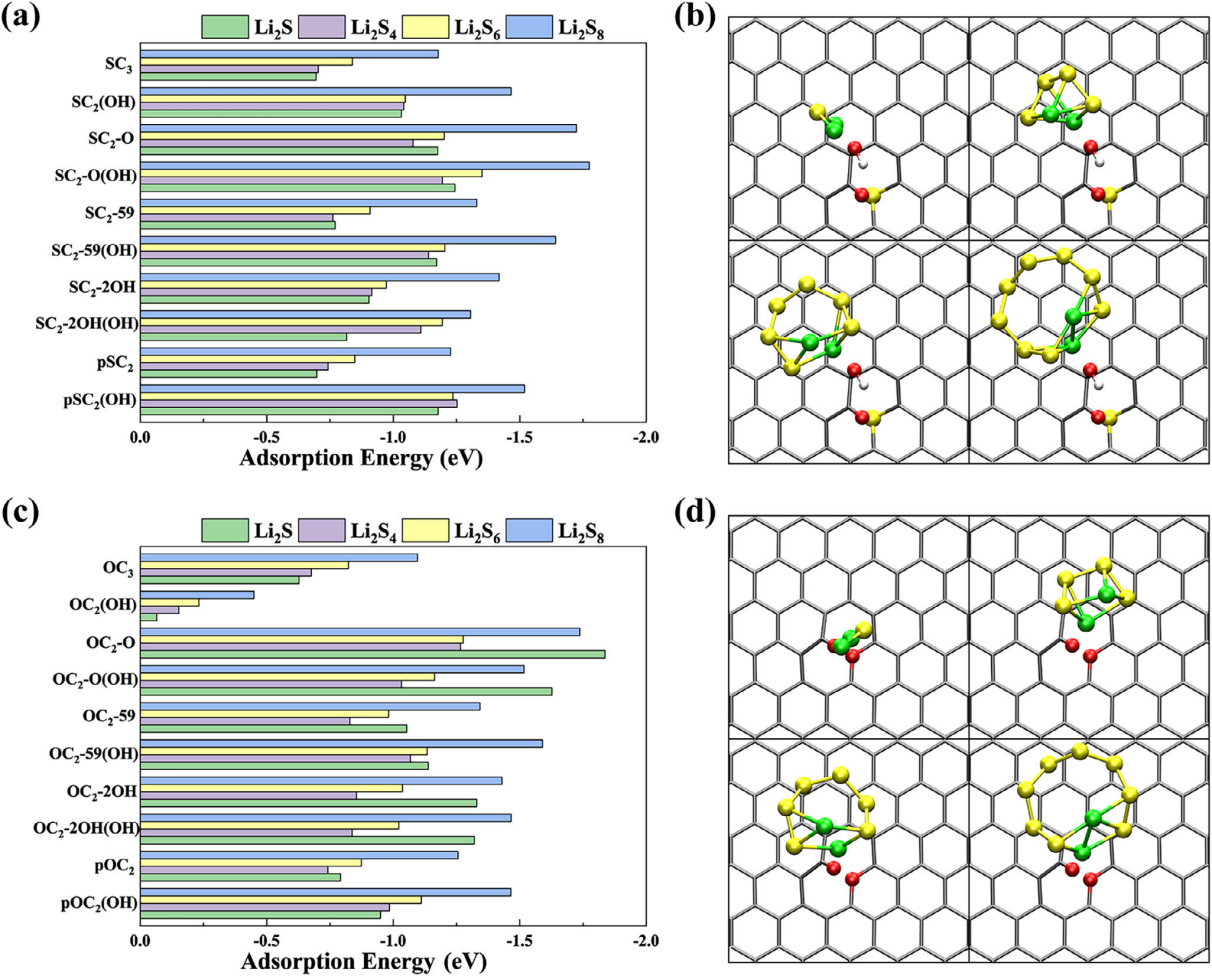
(a) Adsorption energies of Li_2_S, Li_2_S_4_, Li_2_S_6_, and Li_2_S_8_ on S‐bearing defects in SG. Bars are color‐coded: green = Li_2_S, purple = Li_2_S_4_, yellow = Li_2_S_6_, blue = Li_2_S_8_; more negative values (to the right) indicate stronger binding. (b) Representative optimized adsorption geometries of Li_2_S, Li_2_S_4_ and Li_2_S_6_, and Li_2_S_8_ on SC_2_‐O(OH) (top views). (c) Adsorption energies of Li_2_S, Li_2_S_4_, Li_2_S_6,_ and Li_2_S_8_ on O‐bearing defects in SG. (d) Representative optimized adsorption geometries of Li_2_S, Li_2_S_4,_ and Li_2_S_6_, on OC_2_‐O(OH) (top views).

Inspired by previous findings about the ORR/NRR performance of our newly proposed defects shown in Figures 5 and 6, we then explored the mechanism of SRR by evaluating the adsorption behaviors of representative LiPSs (Li_2_S, Li_2_S_4_, Li_2_S_6_, and Li_2_S_8_) on such typical defects, as illustrated in Figures S27–S30. Again, these defect configurations include intrinsic sulfur doping configurations involving oxygen‐containing groups, closely matching experimentally characterized surface chemistry [17, 18, 38, 43, 68] DFT calculations revealed that these in‐plane S‐ and O‐bearing defects exhibit strong interactions with long‐chain LiPSs (Li_2_S_4_, Li_2_S_6_, and Li_2_S_8_), with adsorption energies ranging from −0.63 to −1.84 eV (Figure 7a–c). This demonstrates that LiPSs adsorption strengths on most defects significantly exceed typical solvent‐polysulfide interactions (e.g., 0.9/1.0 eV for DOL/DME electrolytes [69]), confirming robust anchoring capability on SG. Among these defects, SC_2_‐O, SC_2_‐O(OH), OC_2_‐O, and OC_2_‐59(OH) show particularly strong trapping capabilities. For example, the adsorption energies of Li_2_S_4_, Li_2_S_6_, and Li_2_S_8_ on SC_2_‐O(OH)/OC_2_‐O(OH) are −1.19/−1.03, −1.35/−1.16, and −1.77/−1.52 eV, respectively. This is in contrast to the results from the literature, which suggests edged‐S cannot effectively adsorb these long‐chain LiPSs [29]. The corresponding adsorption configurations are illustrated in Figure 7b–d, showing primary bonds formed between Li atoms (from Li_2_S*_n_*) and O atoms (in ─OH groups). Furthermore, OH─ functional group enhances binding affinity to LiPSs of SC_3_, SC_2_‐O, SC_2_‐59, SC_2_‐2OH, and pSC_2_ configurations. Polysulfide binding preferentially occurs near oxygen atoms in these defects, indicating a synergistic mechanism: sulfur defects create reactive sites while adjacent oxygen groups increase local polarity, stabilizing adsorbed polysulfides. Li_2_S adsorption energies were also calculated for comparison with the literature. Most defective structures show adsorption energies ranging from −0.7 to −1.84 eV. The strongest adsorption of Li_2_S is exhibited by OC_2_‐O (−1.84 eV), OC_2_‐O(OH) (−1.63 eV), SC_2_‐O (−1.18 eV) and SC_2_‐O(OH) (−1.24 eV). These values are comparable to those of edge defects reported in Ref [29]. Collectively, these findings demonstrate that basal‐plane defects in SG can provide active sites for SRR and effectively suppress the shuttle effect. Notably, this defect‐centric modeling strategy exhibits broader applicability beyond zigzag‐edged configurations by incorporating both the structural features of SG and the experimental shuttle‐suppression performance.

## 3 Conclusions

In this work, we revisited the long‑standing view that the electrocatalytic activity of SG originates mainly from edge sulfur atoms. Systematic DFT calculations, validated against key experimental observations about suppressing Li dendrite growth and magnetism, revealed that the commonly adopted planar SC_3_@G_in_ model is both thermodynamically and dynamically unstable; the true ground state is an out‑of‑plane SC_3_@G_out_ configuration, which neither binds Li strongly nor carries magnetism. By explicitly introducing S and O atoms into single‑ and double‑vacancy sites—and accounting for the inevitable ─OH/─O surface groups—we constructed a realistic library of twenty basal‑plane defects for SG. Several of these hydroxylated species (e.g., OC_2_‐O(OH) and SC_2_‐O(OH)) exhibit sizeable magnetic moments (0.28–0.55 *µ*_B_) and very low Li‑adsorption energies (up to ∼ −3.1 eV), far exceeding the cohesive energy of bulk Li and thus capable of suppressing dendrite formation. The same defects are also found to be active sites for ORR and NRR. SC_2_‐O(OOH) delivers an ORR overpotential of 0.49 V approaching that of Pt (111), while OC_2_(H) and SC_2_‑O(OH) afford NRR limiting potentials of −1.52 V and −1.69 V, even close to that of edge‑based models. Moreover, they anchor long‑chain polysulfides with adsorption energy as low as −1.8 eV, mitigating the shuttle effect in Li‐S batteries. This resolves the persistent challenge of absent active sites in SG for long‐chain LiPSs adsorption. Overall, realistic S‐doped graphene will inevitably contain both basal‐plane and edge sites, and our results do not exclude possible catalytic contributions from edge sulfur species. Rather, they demonstrate that basal‐plane defects in SG—especially when hydroxylated—can make substantial contributions to magnetism, Li adsorption, and electrocatalytic performance. In this sense, our work extends the conventional edge‐centric picture by highlighting a previously underappreciated family of in‐plane active sites and provides guidance for designing next‐generation graphene‐based electrodes with chalcogen dopants for energy conversion and storage.

## Conflicts of Interest

The authors declare no conflicts of interest.

## Supporting information




**Supporting File**: advs74668‐sup‐0001‐SuppMat.pdf.

## Data Availability

The data that support the findings of this study are available in the supplementary material of this article.
