# Supplementary material for: Beyond the Edge: Basal‐Plane Defects as the Dominant Catalytic Sites in Sulfur‐Doped Graphene
Source: Adv Sci (Weinh). 2026 Mar 2;13(26):e21758. doi: 10.1002/advs.202521758 (PMC13159118; doi:10.1002/advs.202521758)
Supplement: Supplementary file 1 — Supporting File: advs74668‐sup‐0001‐SuppMat.pdf. [file ADVS-13-e21758-s001.pdf]

Beyond the Edge: Basal-Plane Defects as the Dominant Catalytic Sites in Sulfur-Doped Graphene

Xuanhao Yuan, Chenhui Wang, Hui Hu, Hao Cui, Yan Li*, Chengxin Wang*

State Key Laboratory of Optoelectronic Materials and Technologies, School of Materials Science and Engineering, Sun Yat-sen University, Guangzhou 510275, People's Republic of China

* Corresponding Authors

E-mail: liyan266@mail.sysu.edu.cn; wchengx@mail.sysu.edu.cn

Computational Methods

All density-functional theory (DFT) calculations were performed with the Vienna Ab-initio Simulation Package (VASP)[1,2] using the projector-augmented-wave (PAW)[3] method. Exchange–correlation effects were described by the Perdew-Burke-Ernzerhof (PBE)[4] generalized-gradient approximation. A plane-wave kinetic-energy cutoff of 520 eV was used for all calculations. Brillouin-zone integrations employed a Γ-centered 2×2×1 Monkhorst-Pack mesh[5]; convergence tests confirmed that total energies and adsorption energies change by less than 0.02 eV upon increasing the k-mesh for the supercells considered.

Slab models were constructed using single-layer graphene–based supercells with at least 15 Å of vacuum along the surface normal to avoid spurious slab–slab interactions. Lattice parameters were fixed to those optimized for pristine graphene, and all ionic positions were fully relaxed. Two types of supercells were employed depending on the defect model. For the $3SC_3@G_{in}$ and $3SC_3@G_{out}$ configurations, a cell with lattice parameters a = 12.828 Å, b = 9.875 Å and $\alpha = \beta = \gamma = 90°$ was used. For the other defect models, a cell with lattice parameters $a = b$ = 14.808 Å and $\alpha = \beta = 90°$, $\gamma = 120°$ was employed. Spin polarization was included; small initial magnetic moments were assigned to atoms neighboring the defects, and results were confirmed to be independent of the initial spin guess. Electronic and ionic relaxations proceeded until the total-energy change between self-consistent cycles fell below 10^{-5} eV and the residual forces on every atom were below 0.02 eV $Å^{-1}$.

The initial geometries of Li_2S_n (n = 2,4,6,8) clusters were constructed based on previously reported stable configurations.[6–9]

Electrocatalysis calculations

1. Energy definitions

Adsorption energy (used for all adsorbates/intermediates).

$$E_{ads}(X) = E_{tot}(sub + X) - E_{tot}(sub) - E_{tot}(X)$$

Where X can be Li, Li_2S_n, or surface intermediates *H, *OH, *OOH, *NNH, etc. Negative values indicate exothermic binding.

Polysulfides.

$$E_{ads}(Li_2S_n) = E_{tot}(sub + Li_2S_n) - E_{tot}(sub) - E_{tot}(Li_2S_n)$$

Gas-phase reference energies $E_{tot}(X)$ were computed spin-polarized in the same box as the slab for error cancellation.

2. Elementary reaction schemes

("*" denotes a surface site; "(gas)")

ORR

$$O_2 + H^+ + e^- + * \rightarrow *OOH$$

$$*OOH + H^+ + e^- \rightarrow H_2O + *O$$

$$*O + H^+ + e^- \rightarrow *OH$$

$$*OH + H^+ + e^- \rightarrow H_2O + *$$

NRR-distal path

$$*N_2 \rightarrow *NNH \rightarrow *NNH_2 \rightarrow *N \rightarrow *NH \rightarrow *NH_2 \rightarrow NH_3(g)$$

3. Gibbs free-energy corrections

Free-energy changes were obtained as

$$\Delta G = \Delta E + \Delta ZPE - T\Delta S$$

at T = 298 K. Gas-phase entropies were taken from JANAF; vibrational entropies and ZPE from harmonic frequencies. ZPE values were evaluated on $OC_2(OH)$ and transferred to other substrates; spot checks (e.g., on SC_2-O(OH)) confirmed transfer errors $\leq$ a few 10^{-2} eV.

Based on computational hydrogen electrode (CHE) method, we calculated the free energy of an electron-proton pair $H^+ + e^-$ to be $\mu(H^+ + e^-) = 1/2G(H_2)$.[10]

4. Step free energies and (over)potentials

ORR

For 4e- ORR path, the step free energies at U = 0 V are

$$\Delta G_1 = \Delta G_{*OOH} - 4.92$$

$$\Delta G_2 = \Delta G_{*O} - \Delta G_{*OOH}$$

$$\Delta G_3 = \Delta G_{*OH} - \Delta G_{*O}$$

$$\Delta G_4 = -\Delta G_{*OH}$$

Overpotential is calculated by:

$$U_L^{ORR} = \frac{\max(\Delta G_1, G_2, G_3, G_4)}{e}$$

$$\eta_{ORR} = 1.23 - U_L^{ORR}(V)$$

NRR (distal pathway)

We defined the free energy changes of six proton-electron steps along the distal path as $\Delta G_i(0)$ at U = 0 V and calculated the limiting potential by:

$$U_L^{NRR} = -\frac{\max(\Delta G_i(0))}{e} \ (i = 1 \ldots 6)$$

So that a larger U_L^{NRR} (i.e., less negative bias required) indicates better thermodynamic feasibility.

Supplementary Figure

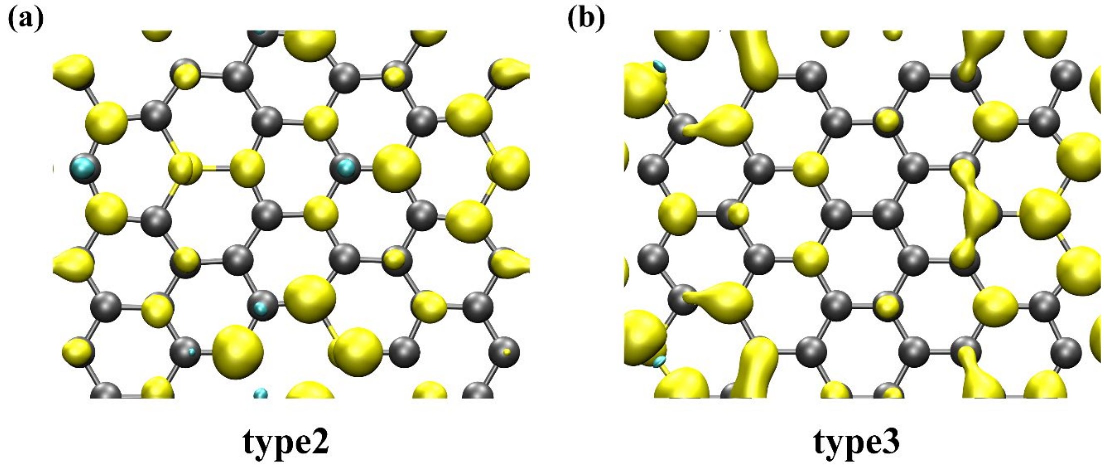

Figure S1. Spin-density isosurfaces for the planar $3SC_3@G_{in}$ models: (a) type2 and (b) type3. The isovalue is 0.005 e Å^{-3}. Yellow lobes highlight regions of largest spin polarization, predominantly around S dopants and nearest-neighbor C atoms. The converged net magnetic moments are 0.266 μ_B (type2) and 0.343 μ_B (type3) per supercell.

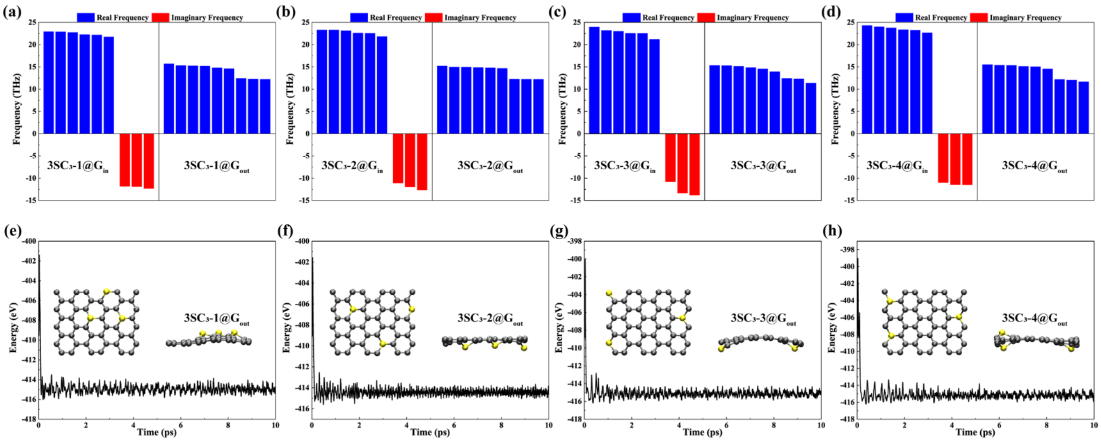

Figure S2. Phonon-mode spectra for four S-doped graphene models of $3SC_3$-n@G_{in} (n = 1, 2, 3, 4) (a–d). Blue bars denote real frequencies; red bars denote imaginary frequencies. All planar S-in-plane configurations ($3SC_3$-n@G_{in}) exhibit imaginary modes, indicating dynamical instability, whereas the corresponding out-of-plane configurations ($3SC_3$-n@G_{out}) are free of imaginary modes and are therefore dynamically stable. (e-h) total-energy profile from a 10 ps AIMD simulation at 300 K for $3SC_3$-n@G_{in}, and snapshots at the end of simulations are presented.

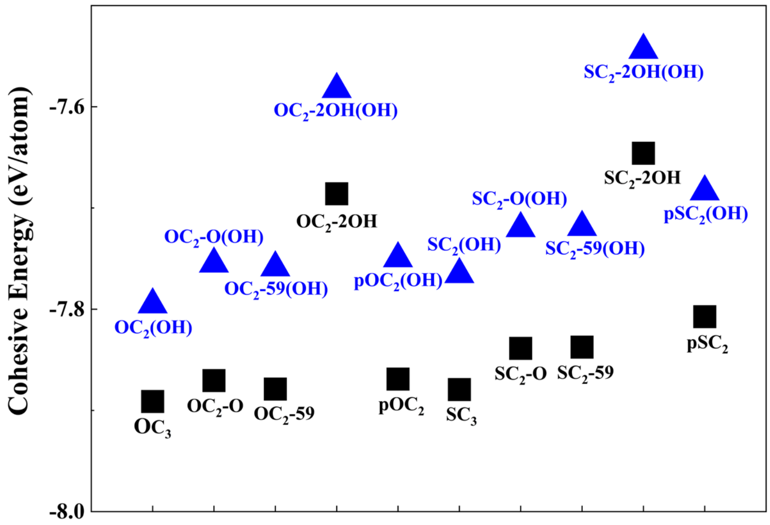

Figure S3 Cohesive energies (eV/atom) of basal-plane defects in SG. Black squares, and blue triangles represent the cohesive energies of typical defects in SG (OC_3, OC_2-O, OC_2-59, OC_2-2OH, pOC_2, SC_3, SC_2-O, SC_2-59, SC_2-2OH, pSC_2) and their OH-functionalized derivatives. More negative values indicate higher thermodynamic stability within this metric. Cohesive energies are calculated as $E_{coh} = \frac{E_{tot} - \Sigma_i n_i E_i}{N}$, where E_{tot} is the total energy of the defective system, n_i is the number of atoms of atoms of type i, and E_i is the reference energy of each isolated atom, and N is the total number of atoms in the system.

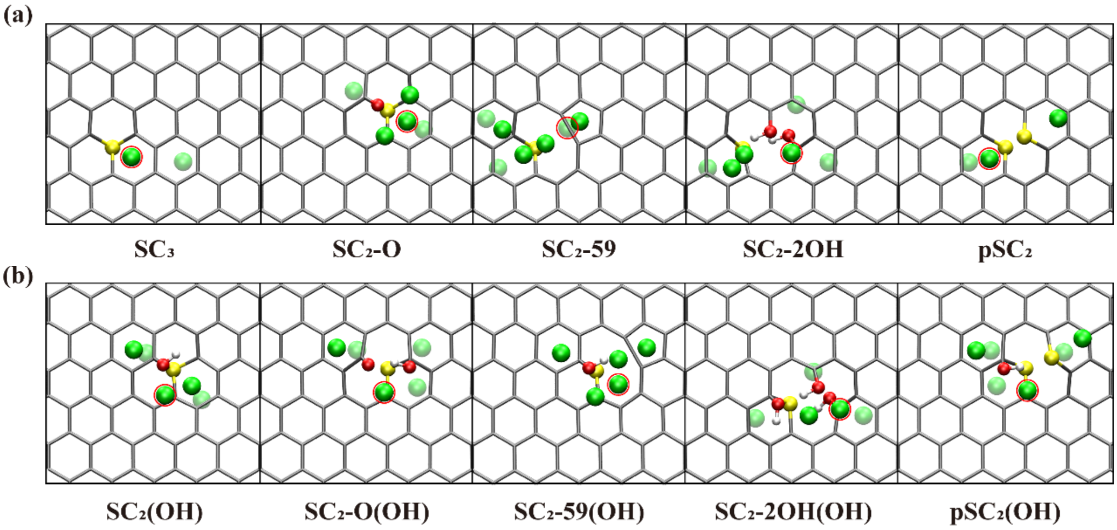

Figure S4. Possible Li adsorption sites on S-bearing basal-plane defects. (a) Intrinsic defects: SC_3, SC_2-O, SC_2-59, SC_2-2OH and pSC_2. (b) Hydroxylated counterparts: SC_2(OH), SC_2-O(OH), SC_2-59(OH), SC_2-2OH(OH) and pSC_2(OH). Solid green spheres mark Li above the graphene plane; translucent green spheres indicate Li below

the plane. The red circles highlight the most strongly bound Li site for each defect. Grey = C, yellow = S, red = O, white = H.

Figure S5.

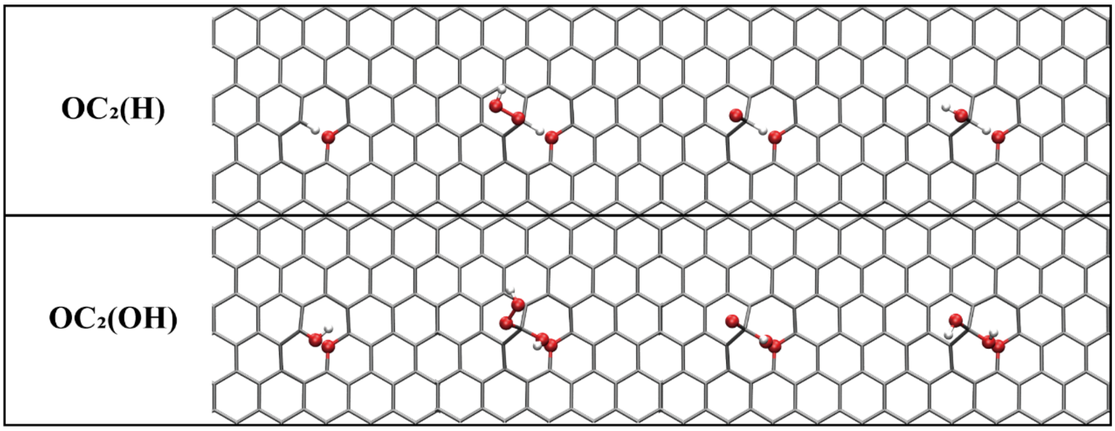

Figure S5. ORR reaction intermediates on H- and OH- functionalized OC_3.

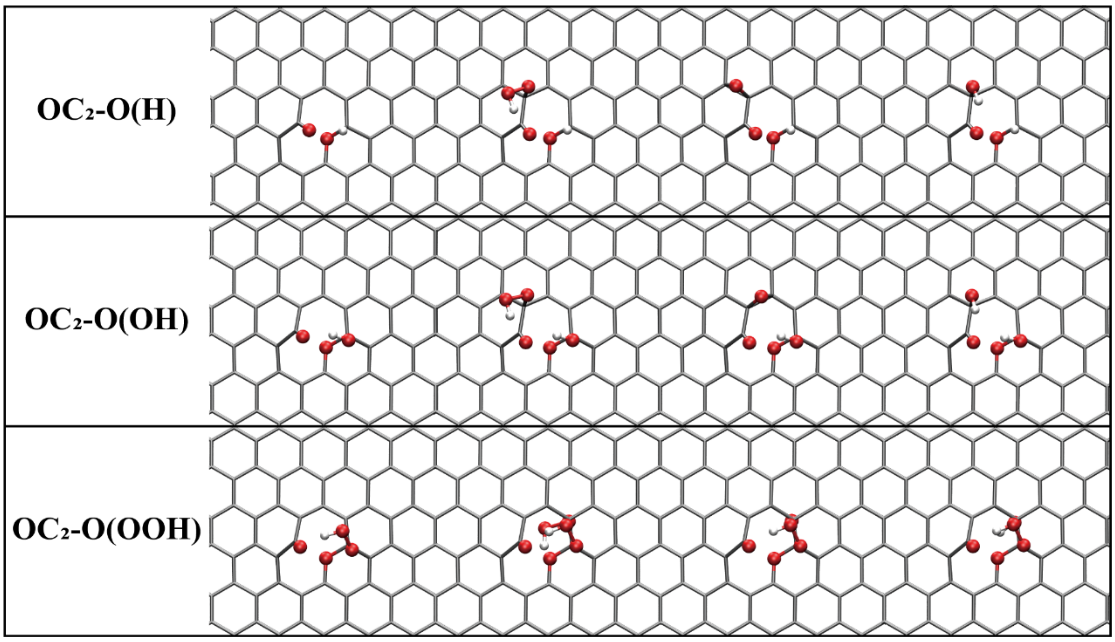

Figure S6. ORR reaction intermediates on H-, OH- and OOH-functionalized OC_2-O.

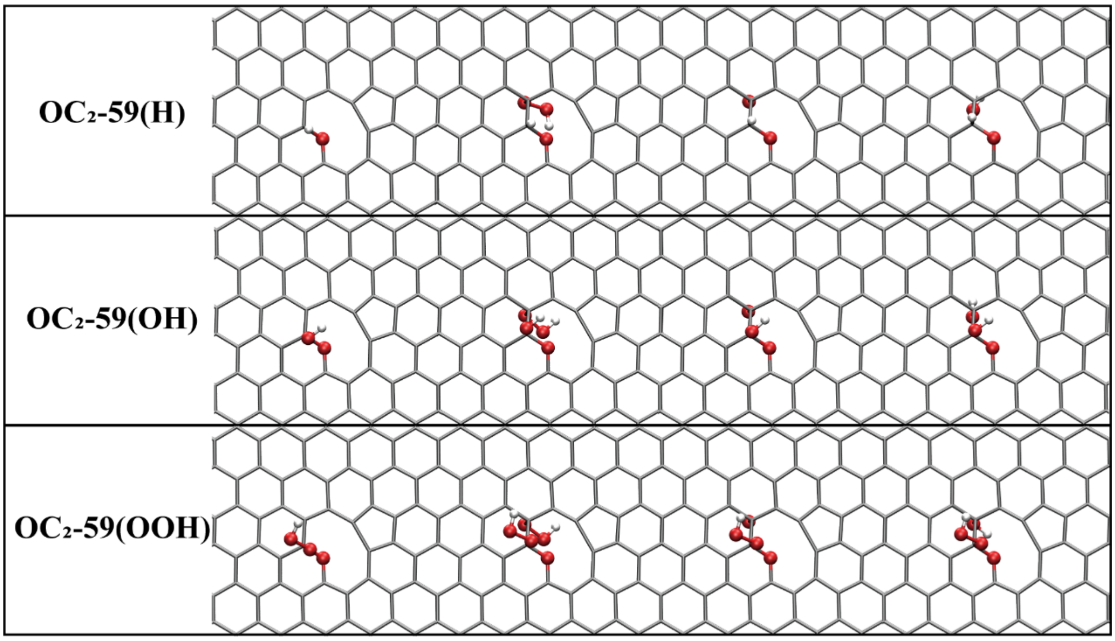

Figure S7. ORR reaction intermediates on H-, OH- and OOH-functionalized OC_2-59.

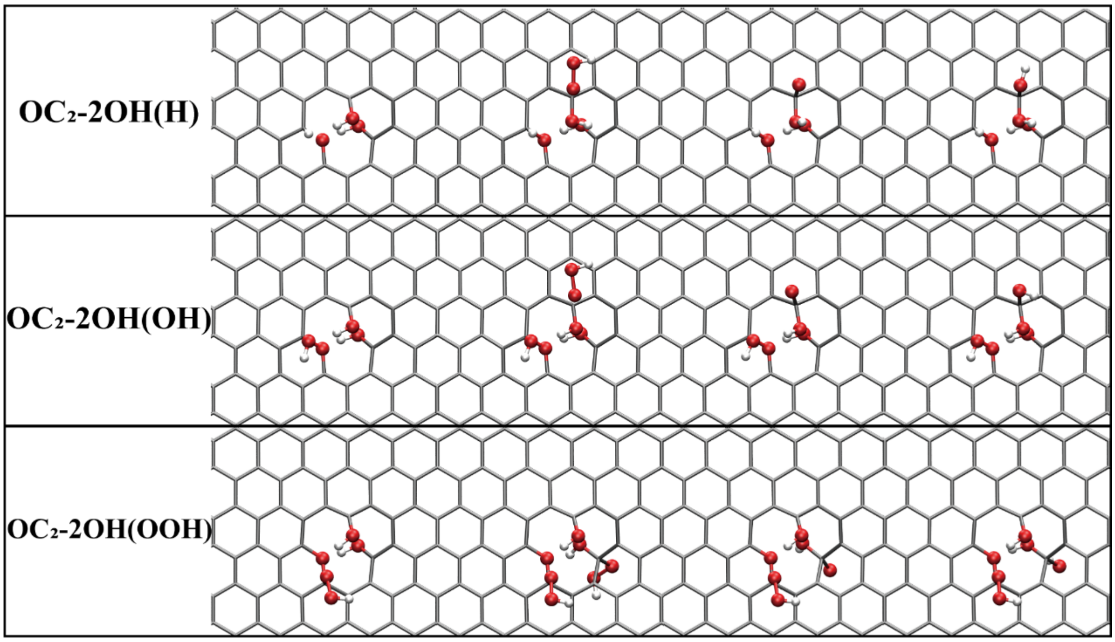

Figure S8. ORR reaction intermediates on H-, OH- and OOH-functionalized OC_2-2OH.

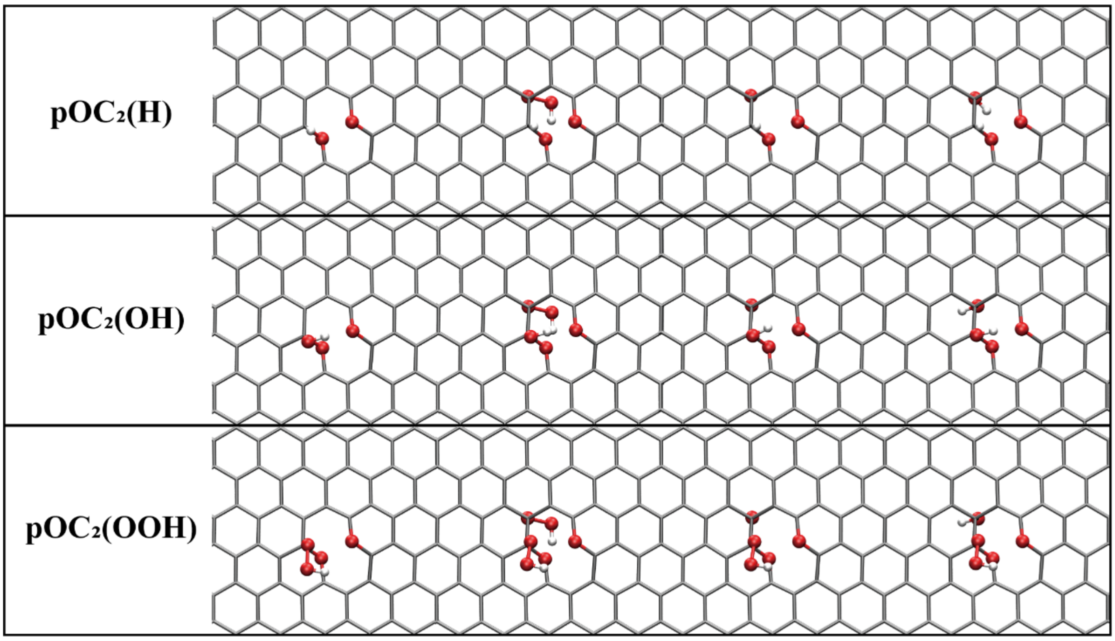

Figure S9. ORR reaction intermediates on H-, OH- and OOH-functionalized pOC_2.

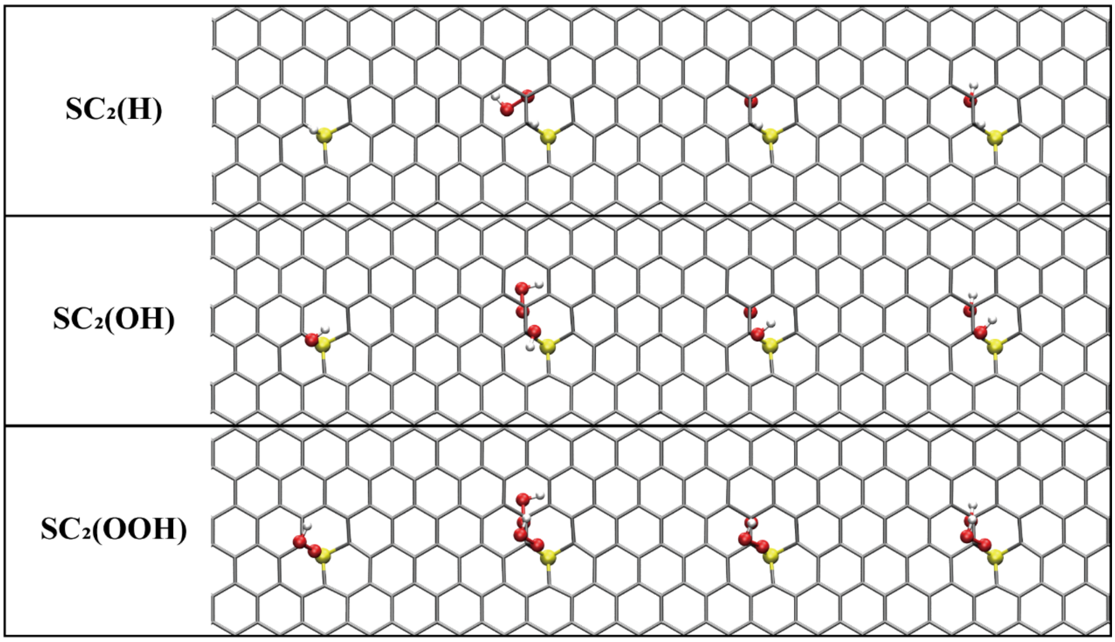

Figure S10. ORR reaction intermediates on H-, OH- and OOH-functionalized SC_3.

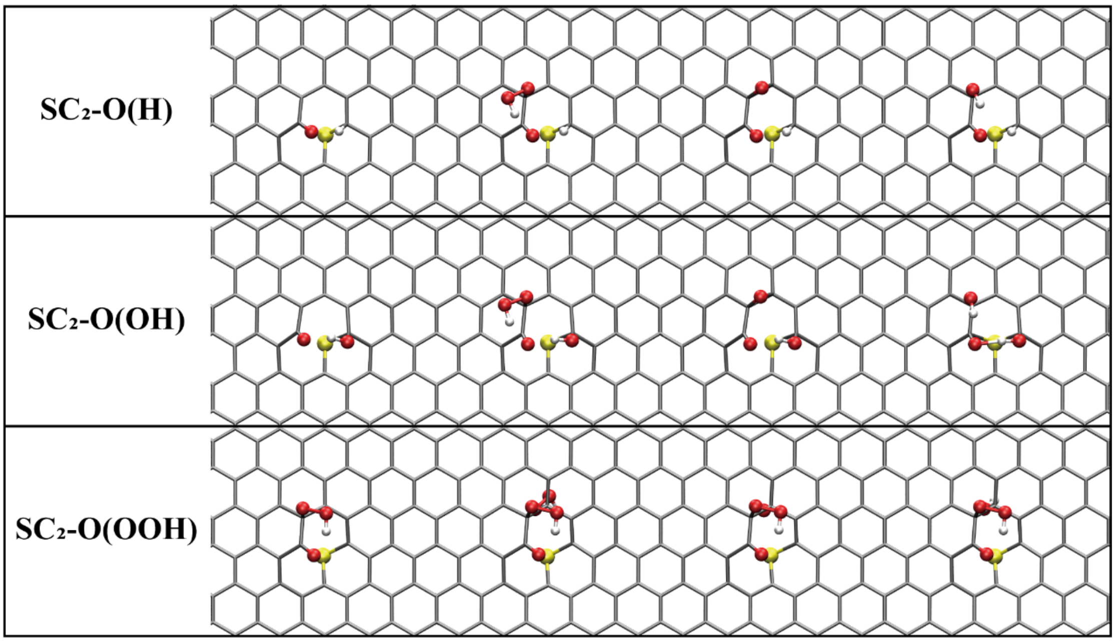

Figure S11. ORR reaction intermediates on H-, OH- and OOH-functionalized SC_2-O.

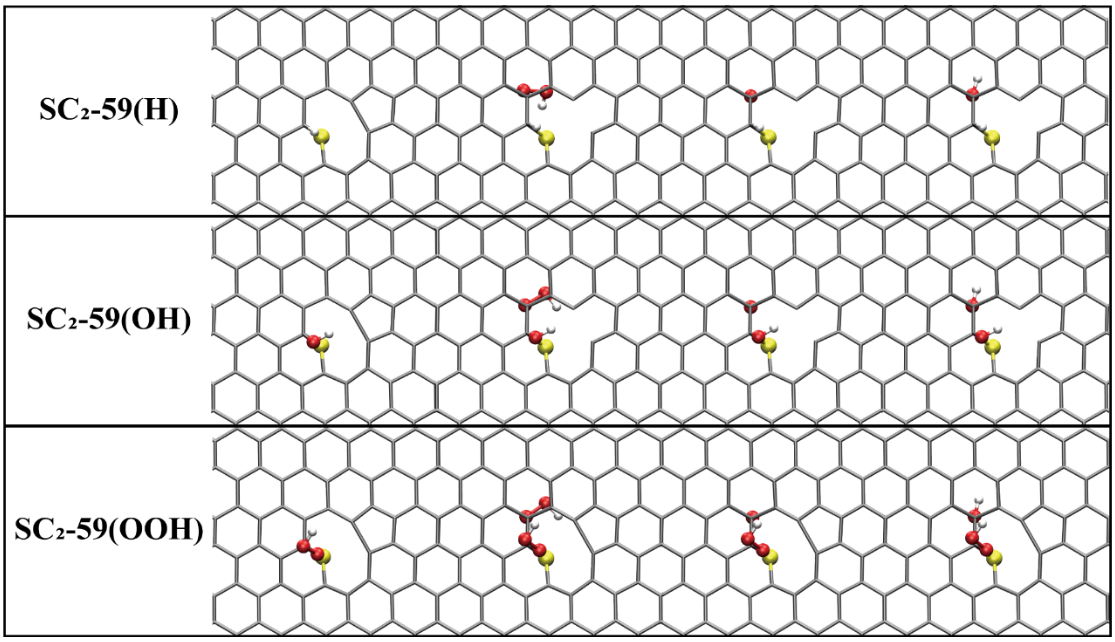

Figure S12. ORR reaction intermediates on H-, OH- and OOH-functionalized SC_2-59.

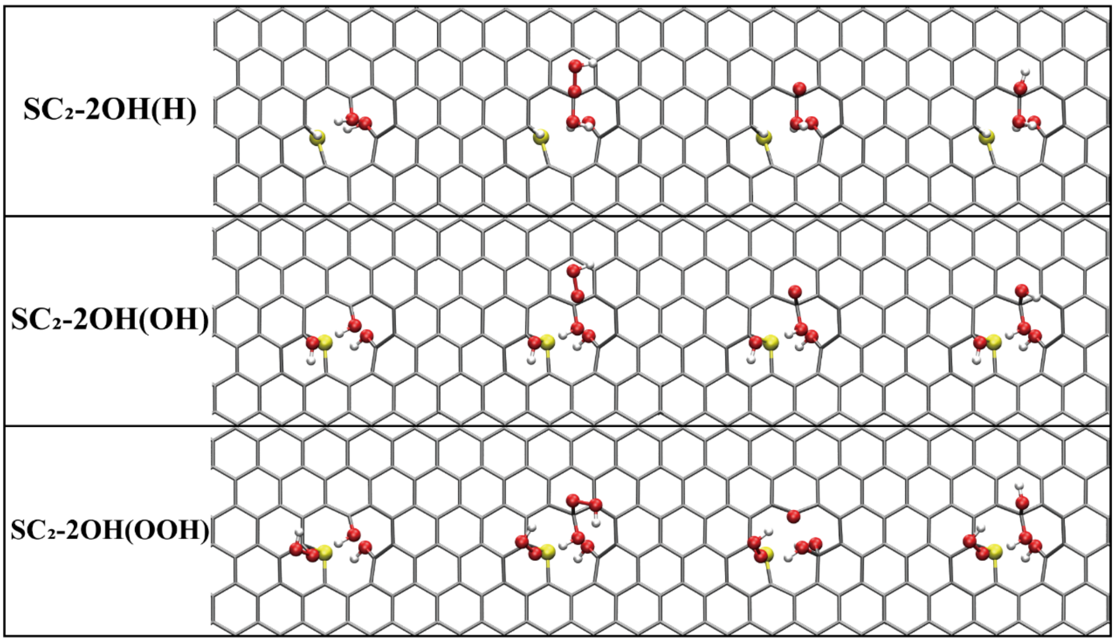

Figure S13. ORR reaction intermediates on H-, OH- and OOH-functionalized SC_2-2OH.

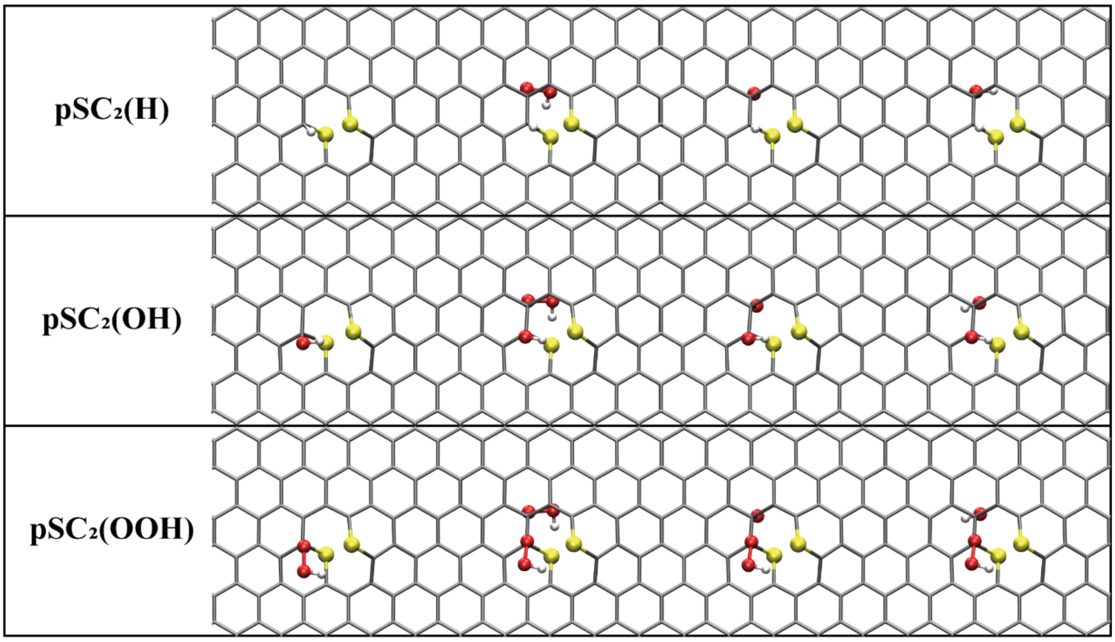

Figure S14. ORR reaction intermediates on H-, OH- and OOH-functionalized pSC_2.

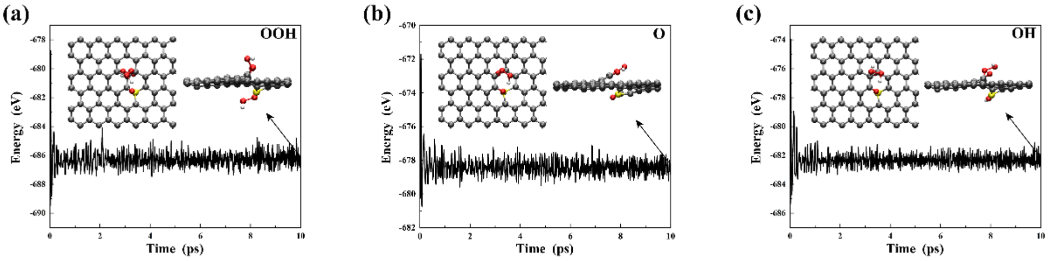

Figure S15. AIMD results for OOH*, O*, and OH* intermediates on the SC_2-O(OOH) defect site at 300 K, confirming their thermal stability without desorption or reconstruction.

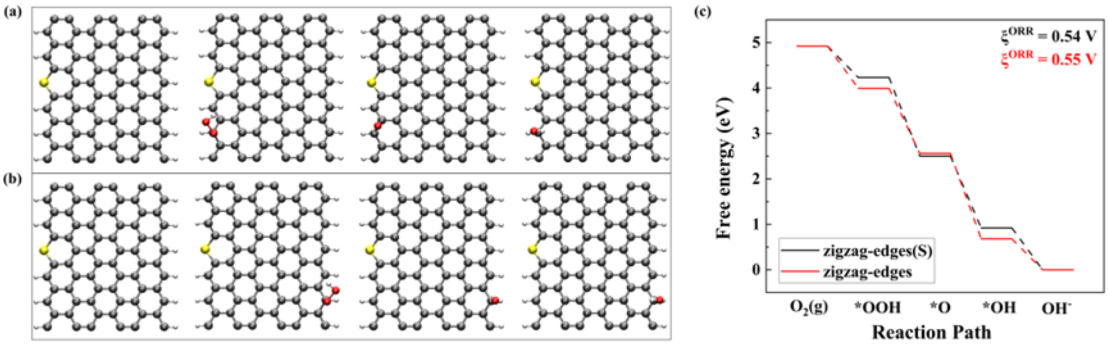

Figure S16. Optimized adsorption configurations of ORR intermediates on zigzag-edge sulfur-doped graphene. (a) Sulfur doped on the same side of the reaction site. (b) The opposite side without sulfur doping. For each case, the adsorption geometries of *OOH, *O, and *OH intermediates are shown, illustrating the reaction pathways on zigzag-edge models used for benchmarking against basal-plane defects.

General note (Figures S17-S26). Each figure displays relaxed geometries of reaction intermediates along the distal NRR pathway on a given basal-plane defect. From left to right: *NNH, $*NNH_2$, *N, *NH, $*NH_2$, and $*NH_3$(g). Colors: grey = C, red = O, yellow = S, blue = N, white = H.

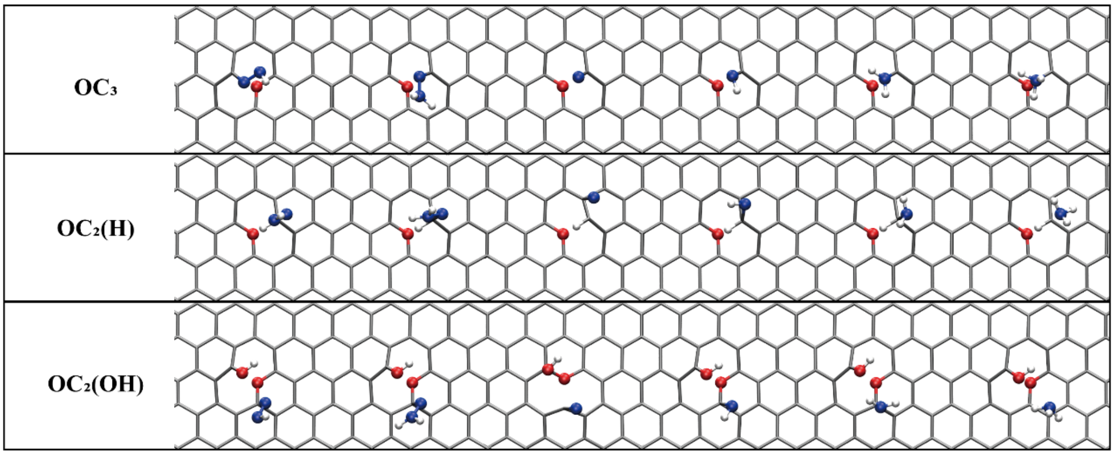

Figure S17. NRR reaction intermediates on pristine OC_3, H-functionalized OC_2(H) and

OH-functionalized OC_2(OH).

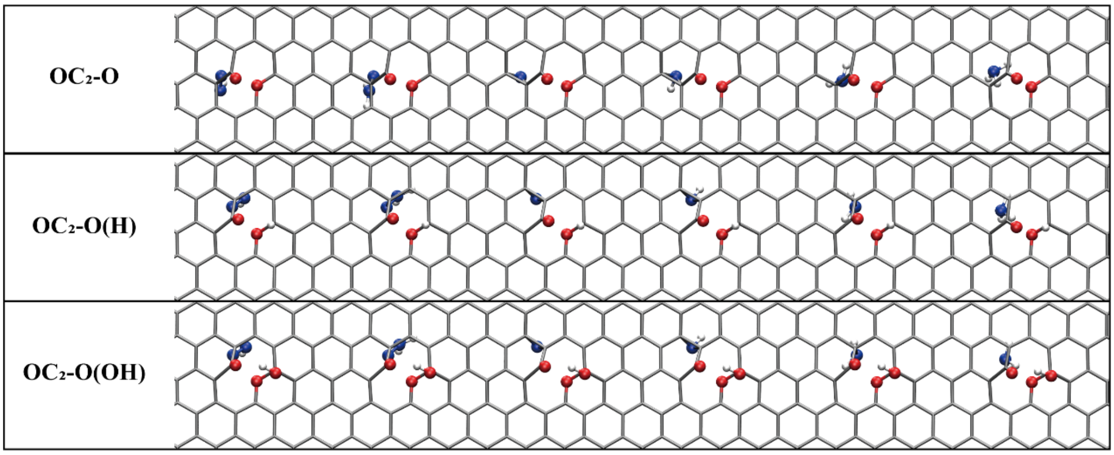

Figure S18. NRR reaction intermediates on pristine OC_2-O, H-functionalized OC_2-O(H) and OH-functionalized OC_2-O(OH).

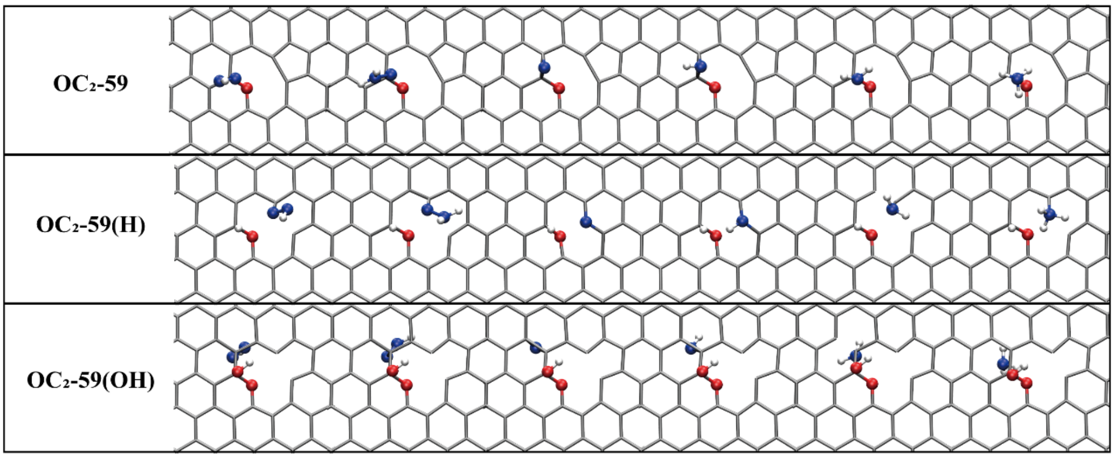

Figure S19. NRR reaction intermediates on pristine OC_2-59, H-functionalized OC_2-59(H) and OH-functionalized OC_2-59(OH).

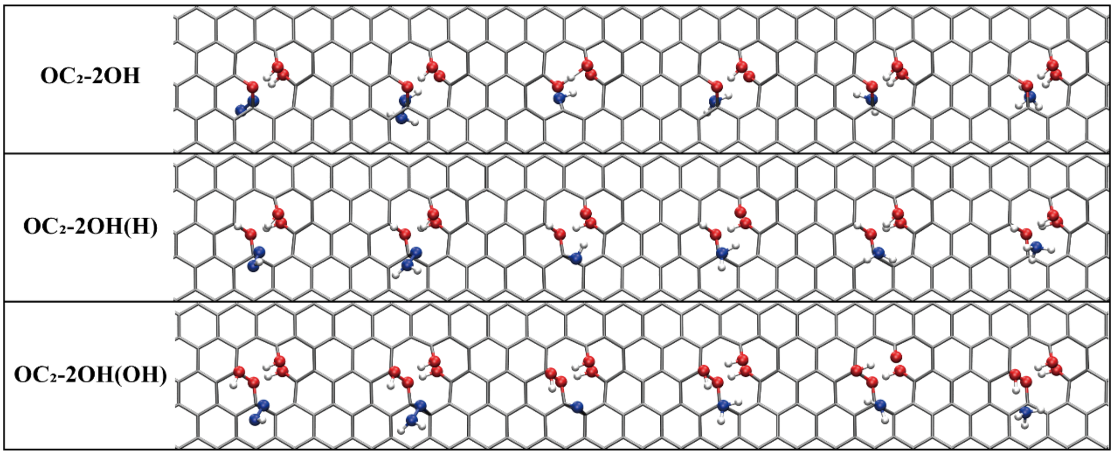

Figure S20. NRR reaction intermediates on pristine OC_2-2OH, H-functionalized OC_2-2OH(H) and OH-functionalized OC_2-2OH(OH).

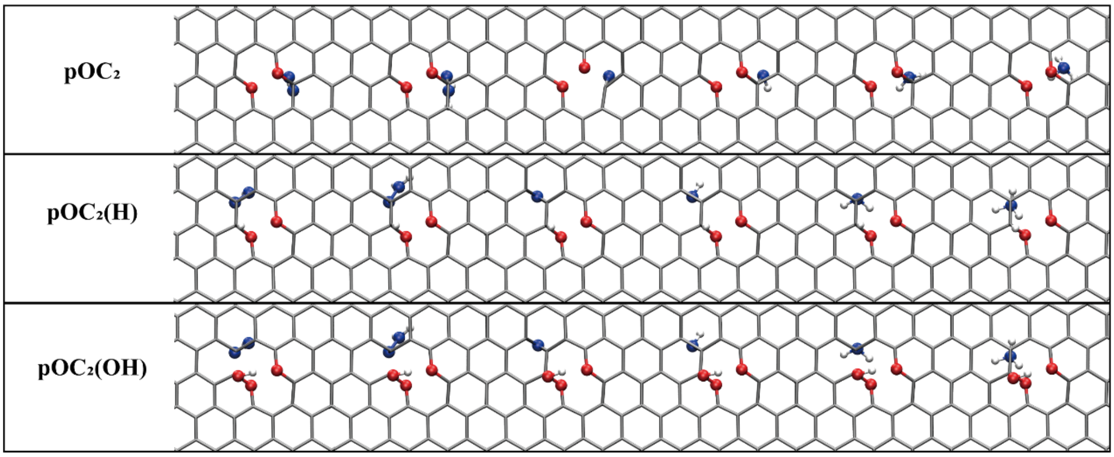

Figure S21. NRR reaction intermediates on pristine pOC_2, H-functionalized pOC_2(H) and OH-functionalized pOC_2(OH).

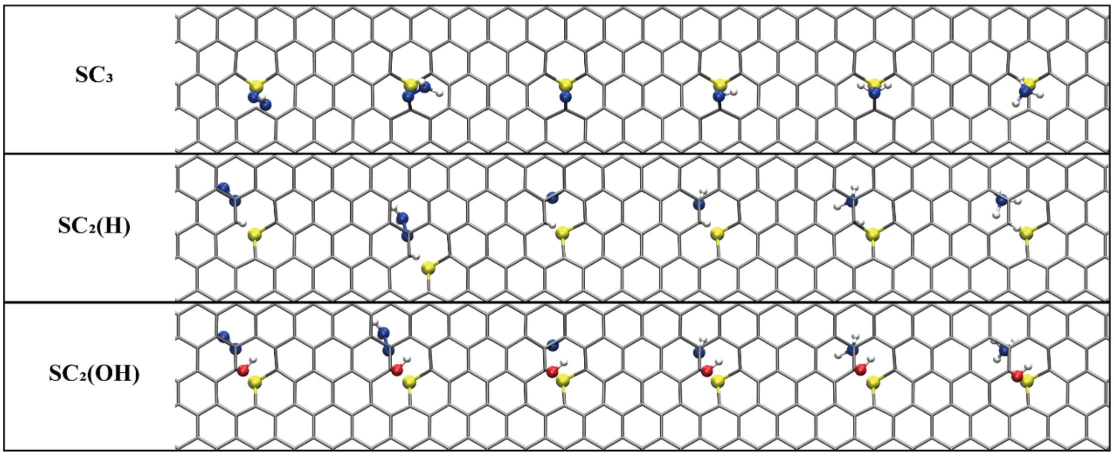

Figure S22. NRR reaction intermediates on pristine SC_3, H-functionalized SC_2(H) and OH-functionalized SC_2(OH).

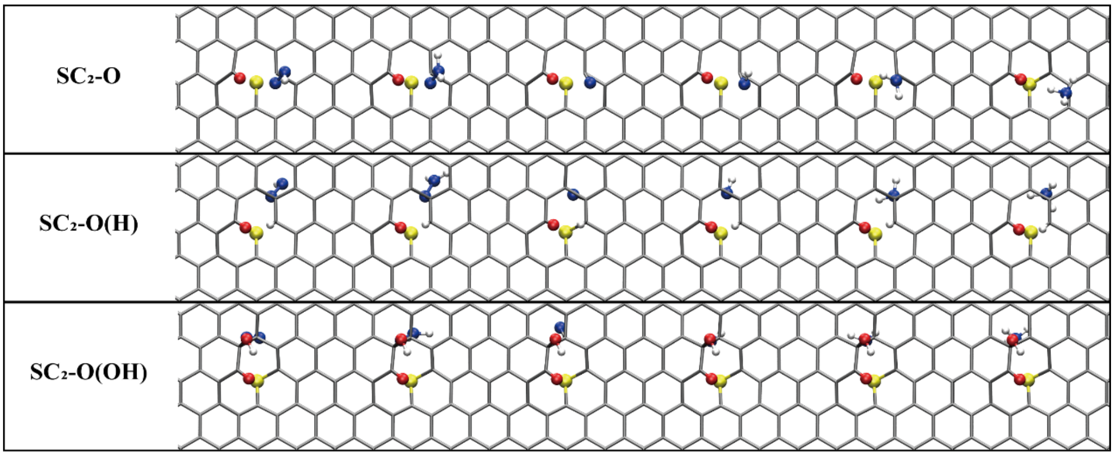

Figure S23. NRR reaction intermediates on pristine SC_2-O, H-functionalized SC_2-O(H) and OH-functionalized SC_2-O(OH).

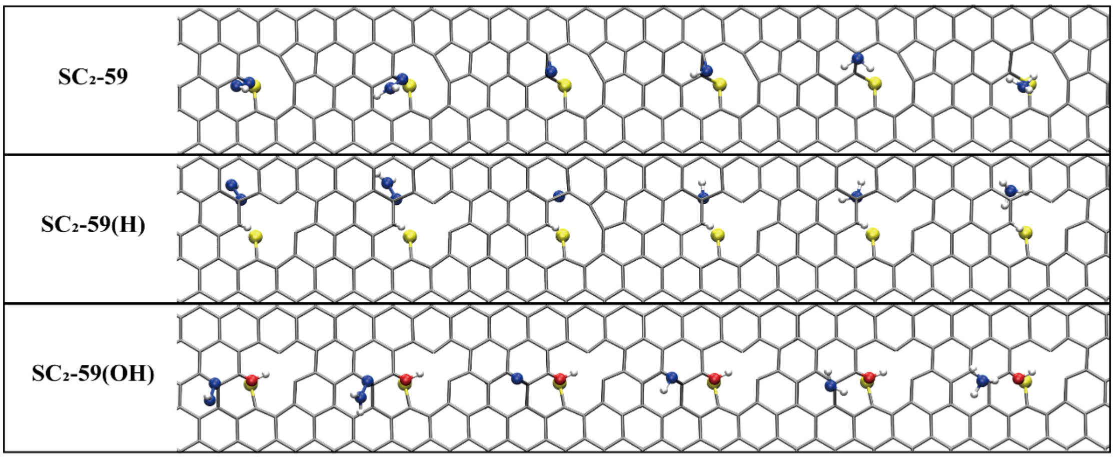

Figure S24. NRR reaction intermediates on pristine SC_2-59, H-functionalized SC_2-59(H) and OH-functionalized SC_2-59(OH).

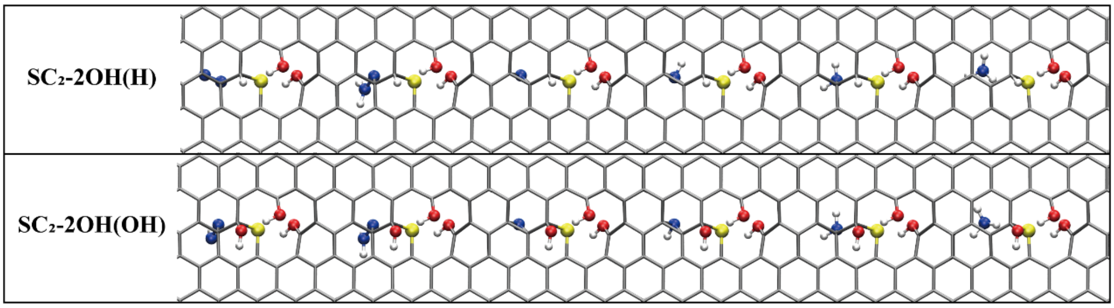

Figure S25. NRR reaction intermediates on pristine SC_2-2OH, H-functionalized SC_2-2OH(H) and OH-functionalized SC_2-2OH(OH).

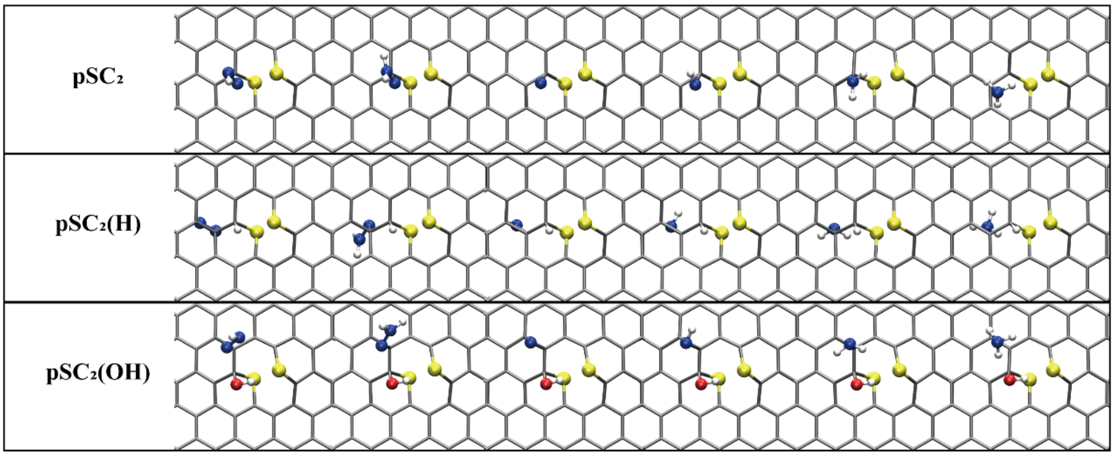

Figure S26. NRR reaction intermediates on pristine pSC_2, H-functionalized pSC_2(H)

and OH-functionalized $pSC_2(OH)$.

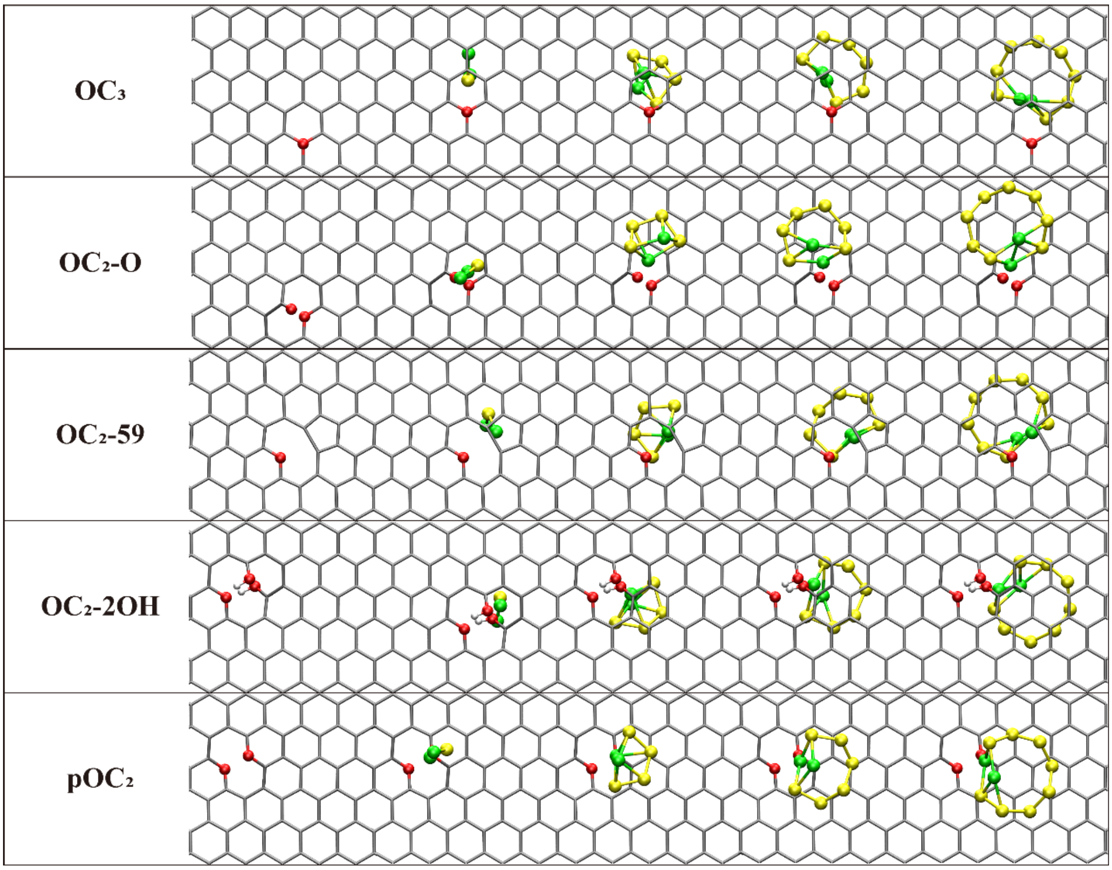

Figure S27. Optimized adsorption geometries of lithium polysulfides on O-bearing basal-plane defects. Rows (top to bottom): OC_3, OC_2-O, OC_2-59, OC_2-2OH, pOC_2. Columns (left to right): Li_2S, Li_2S_4, Li_2S_6, Li_2S_8. For each case, the top-view configuration corresponding to the lowest-energy is shown. These snapshots correspond to the adsorption energies reported in Table S5.

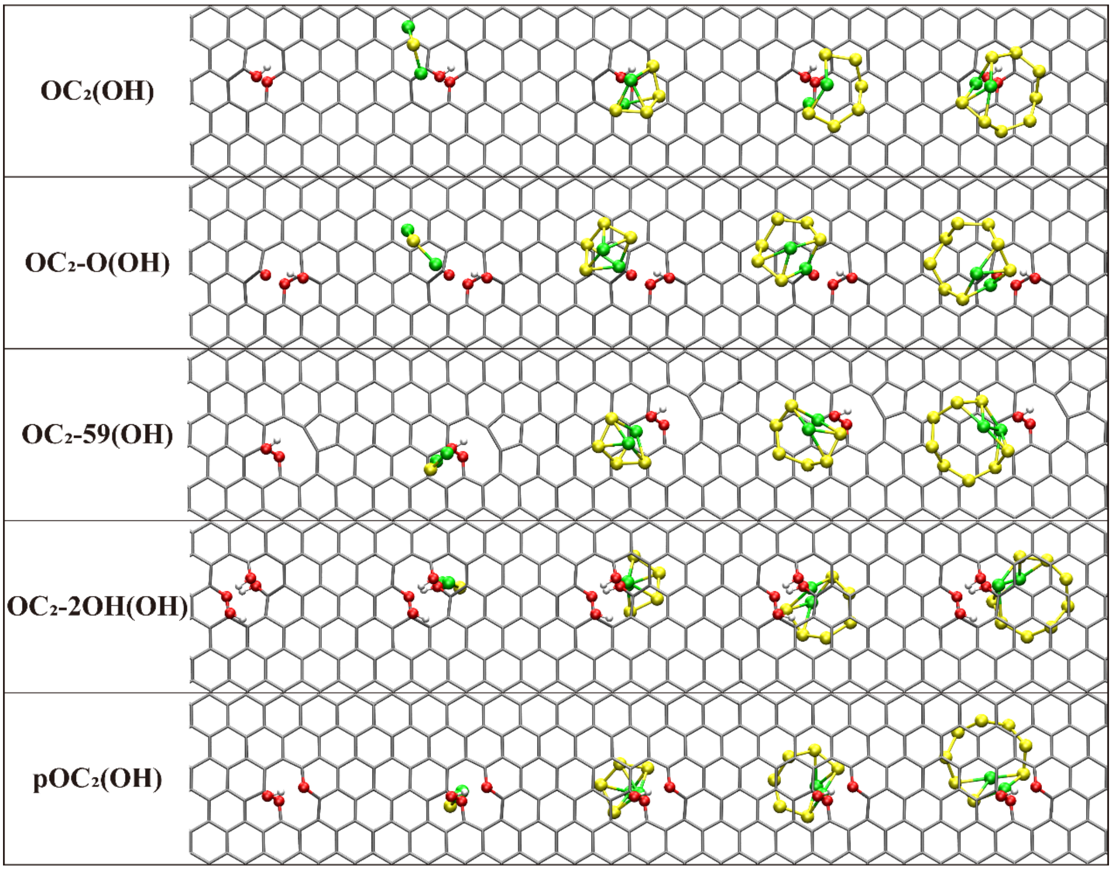

Figure S28. Optimized adsorption geometries of lithium polysulfides on hydroxylated O-bearing basal-plane defects. Rows (top to bottom): $OC_2(OH)$, OC_2-O(OH), OC_2-59(OH), OC_2-2OH(OH), $pOC_2(OH)$. Columns (left to right): Li_2S, Li_2S_4, Li_2S_6, Li_2S_8. Each panel shows the lowest-energy top-view structure after full relaxation.

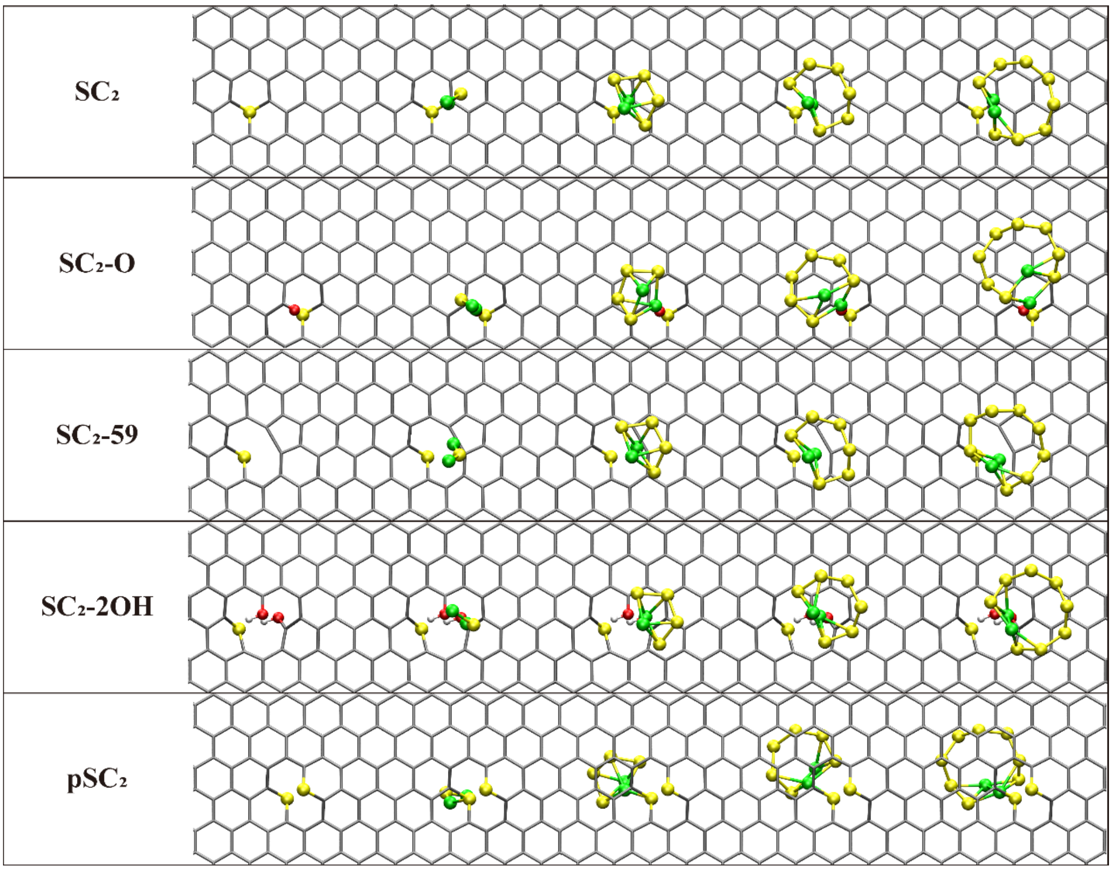

Figure S29. Optimized adsorption geometries of lithium polysulfides on S-bearing basal-plane defects. Rows (top to bottom): SC_2, SC_2-O, SC_2-59, SC_2-2OH, pSC_2. Columns (left to right): Li_2S, Li_2S_4, Li_2S_6, Li_2S_8. Lowest-energy top-view geometries are shown after full relaxation. Energetics are summarized in Table S5.

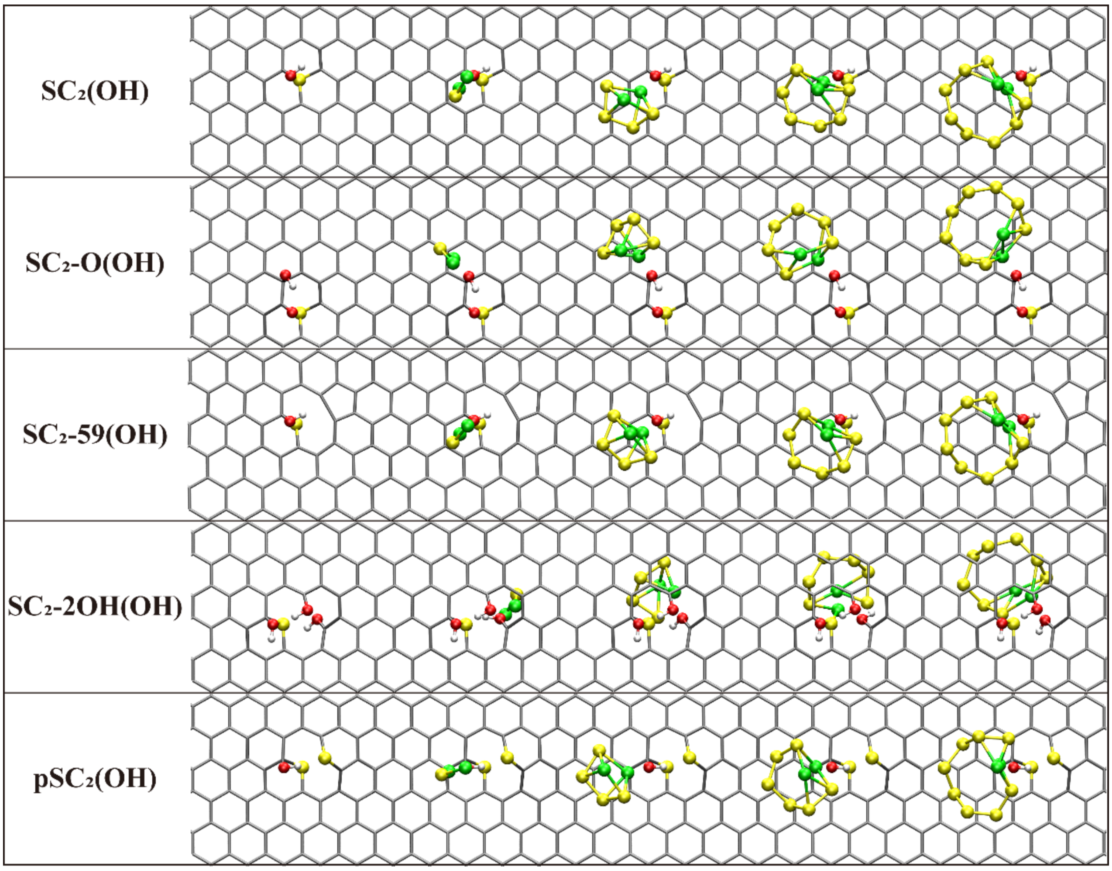

Figure S30. Optimized adsorption geometries of lithium polysulfides on hydroxylated S-bearing basal-plane defects. Rows (top to bottom): SC_2(OH), SC_2-O(OH), SC_2-59(OH), SC_2-2OH(OH), pSC_2(OH). Columns (left to right): Li_2S, Li_2S_4, Li_2S_6, Li_2S_8. Lowest-energy top-view structures after full relaxation are shown. See Table S5 for corresponding adsorption energies.

Supplementary Tables

Table S1. OH binding energies (eV) on oxygen- and sulfur-containing basal-plane defects in SG.

Substrates	Dopant (O/S)	Final Structure	OH Binding Energy (eV)
Graphene		Graphene (OH)	-1.23
OC_3	O	OC_2(OH)	-3.86
OC_2(O)	O	OC_2(O)(OH)	-2.12
OC_2-59	O	OC_2-59(OH)	-1.97
OC_2-2OH	O	OC_2-2OH(OH)	-2.49
pOC_2	O	pOC_2(OH)	-2.08
SC_3	S	SC_2(OH)	-2.37
SC_2(O)	S	SC_2(O)(OH)	-1.93
SC_2-59	S	SC_2-59(OH)	-2.14
SC_2-2OH	S	SC_2-2OH(OH)	-2.53
pSC_2	S	pSC_2(OH)	-1.55

Table S2. Single-Li adsorption tests on basal-plane defects. Adsorption energy E_{ads}(Li) (eV) is calculated by $E_{ads}(Li) = E_{(sub+Li)} - E_{sub} - E_{Li(single)}$. One Li atom per supercell. "E_{ads}-1~10" correspond to distinct initial Li adsorption sites. (More-negative E_{ads}(Li) indicates stronger binding to the substrate).

**Substrates**	E_{ads}-1	E_{ads}-2	E_{ads}-3	E_{ads}-4	E_{ads}-5	E_{ads}-6	E_{ads}-7	E_{ads}-8	E_{ads}-9	E_{ads}-10
OC_3	**-1.386**	-0.700								
OC_2(OH)	-0.795	**-1.317**	-1.317	-1.317	-1.045	-1.287	-1.287	-0.808	-1.286	-1.317
OC_2(O)	-1.673	-1.173	**-2.631**	-1.030						
OC_2(O)(OH)	-2.744	**-3.077**	-2.718							
OC_2-59	-1.167	-1.490	**-1.947**							
OC_2-59(OH)	-2.485	**-2.954**	-2.091	-2.085	-1.888	-2.673	-2.671			
OC_2-2OH	-2.153	-2.157	-1.017	-1.439	-1.851	**-2.196**				
OC_2-2OH(OH)	0.881	-1.881	0.155	-2.383	-1.999	**-2.854**				
pOC_2	-1.589	**-1.723**	-1.606							
pOC_2(OH)	-1.767	-3.258	-2.576	-3.258	-2.490	**-3.378**	-3.380	-3.379	-2.428	-2.131
SC_3	-1.555	**-1.733**								
SC_2(OH)	-2.228	-2.512	-2.508	-2.510	-2.519	**-2.686**	-2.064	-2.654		
SC_2(O)	-2.986	-2.966	-2.965	**-2.986**	-1.868	-2.200	-2.203	-2.204		
SC_2(O)(OH)	**-2.966**	-1.987	-2.127	-2.125	-1.938	-2.965				
SC_2-59	-1.778	-1.682	-2.121	-2.000	-1.794	-1.657	**-2.186**	-2.182		
SC_2-59(OH)	-2.402	-2.685	**-2.775**	-2.235						
SC_2-2OH	-2.234	-2.233	-2.233	-2.233	-1.475	-1.667	-2.214	**-2.245**	-2.209	-1.650
SC_2-2OH(OH)	-2.541	-2.541	-2.552	-1.933	-2.037	-2.443	**-2.858**	-2.439	-1.866	
pSC_2	-1.835	-2.077	**-2.081**							
pSC_2(OH)	-2.442	-2.443	-2.441	-2.442	-2.219	-2.162	-2.535	3.113	-2.581	**-2.640**

Table S3. Adsorption free energies of reaction intermediates of OOH, O and OH and overpotentials for ORR and OER on functionalized five prototypes of S/O-containing defects, which are unavoidably generated during the synthesis of heteroatom doped rGO or during the process of ORR.

Substrates	ΔG_{*OOH} (eV)	ΔG_{*O} (eV)	ΔG_{*OH} (eV)	ξ^{ORR} (V)
OC_2(H)	4.03	2.49	0.56	0.67
OC_2(OH)	4.24	2.53	0.91	0.56
OC_2-O(H)	4.60	2.93	1.21	0.91
OC_2-O(OH)	4.56	2.95	1.24	0.87
OC_2-O(OOH)	4.47	2.79	1.04	0.78
OC_2-59(H)	4.38	2.73	1.00	0.69
OC_2-59(OH)	4.44	2.74	1.02	0.75
OC_2-59(OOH)	4.55	2.86	1.09	0.86
OC_2-2OH(H)	4.97	3.03	1.47	1.28
OC_2-2OH(OH)	4.67	2.98	1.16	0.98
OC_2-2OH(OOH)	4.87	3.25	1.51	1.19
pOC_2(H)	4.21	2.69	0.85	0.52
pOC_2(OH)	4.27	2.69	1.01	0.58
pOC_2(OOH)	4.29	2.66	1.00	0.60
SC_2(H)	4.50	2.31	1.18	0.81
SC_2(OH)	4.67	2.24	1.19	0.98
SC_2(OOH)	4.74	2.37	1.34	1.05
SC_2-O(H)	4.57	2.95	1.19	0.88
SC_2-O(OH)	4.42	2.83	1.08	0.73
SC_2-O(OOH)	4.15	2.32	0.76	0.47
SC_2-59(H)	4.63	2.70	0.97	0.94
SC_2-59(OH)	4.61	2.57	1.09	0.92
SC_2-59(OOH)	4.70	2.70	1.17	1.01
SC_2-2OH(H)	4.31	2.35	0.84	0.63
SC_2-2OH(OH)	4.71	3.05	1.21	1.02
SC_2-2OH(OOH)	4.51	2.36	1.26	0.82
pSC_2(H)	4.62	2.78	1.19	0.93
pSC_2(OH)	4.76	2.71	1.24	1.07
pSC_2(OOH)	4.69	2.64	1.17	1.00

Table S4. Stepwise free-energy changes (ΔG, eV) for NRR on various defects in SG, with and without H/OH functionalization. Values are reported for each elementary step along the distal pathway ($*NNH \rightarrow *NNH_2 \rightarrow *N \rightarrow *NH \rightarrow *NH_2 \rightarrow *NH_3$). All values are given per supercell, calculated within the computational hydrogen electrode (CHE) framework at 298 K and pH = 0.

Substrates	ΔG*NNH (eV)	$\Delta G*NNH_2$ (eV)	ΔG*N (eV)	ΔG*NH (eV)	$\Delta G*NH_2$ (eV)	$\Delta G*NH_3$ (eV)
OC_3	0.767	-1.146	-0.041	-1.586	-0.376	0.889
OC_2(H)	1.519	0.191	-0.216	-1.048	-1.648	-0.431
OC_2(OH)	2.509	0.085	-1.187	-0.513	-1.315	-1.081
OC_2-O	2.642	0.136	-0.292	-1.552	-1.101	-1.250
OC_2-O(H)	2.132	0.171	0.070	-1.597	-1.406	-0.833
OC_2-O(OH)	2.044	0.164	0.081	-1.585	-1.448	-0.704
OC_2-59	2.685	-0.081	-0.173	-1.587	-1.171	-1.128
OC_2-59(H)	2.055	0.147	0.048	-1.571	-1.534	-0.835
OC_2-59(OH)	2.063	0.767	-0.058	-1.671	-1.165	-1.386
OC_2-2OH(H)	2.561	-0.399	0.019	-1.532	-1.479	-0.811
OC_2-2OH(OH)	2.907	-0.267	-0.336	-2.423	-0.125	-1.635
pOC_2	2.631	0.004	-0.488	-1.250	-1.216	-1.146
pOC_2(H)	1.928	0.139	0.026	-1.584	-1.464	-0.482
pOC_2(OH)	1.918	0.162	0.047	-1.592	-1.478	-0.495
SC_3	2.332	-1.216	-0.031	-1.582	-0.617	-0.596
SC_2(H)	2.235	0.151	-1.011	-0.431	-1.636	-0.991
SC_2(OH)	2.232	0.133	-1.025	-0.445	-1.613	-1.018
SC_2-O	3.031	-0.228	-0.318	-1.348	-1.267	-1.687
SC_2-O(H)	2.266	0.188	0.160	-1.540	-1.580	-1.192
SC_2-O(OH)	1.692	0.084	0.174	-1.379	-1.887	-0.476
SC_2-59	2.520	0.071	-0.442	-1.386	-1.174	-1.341
SC_2-59(H)	2.109	0.221	0.030	-1.257	-1.877	-0.934
SC_2-59(OH)	2.465	0.127	-0.055	-1.585	-1.353	-1.330
SC_2-2OH(H)	2.531	-0.385	0.246	-1.387	-1.918	-1.105
SC_2-2OH(OH)	2.452	0.168	-0.114	-1.428	-1.377	-1.425
pSC_2	2.926	-0.154	-0.368	-1.233	-1.007	-1.868
pSC_2(H)	2.344	-0.004	0.029	-1.495	-1.510	-1.071

$pSC_2(OH)$	2.175	0.225	-0.078	-1.538	-1.436	-1.065

Table S5. Adsorption energies (E_{ads}, eV) of lithium polysulfides on representative substrates. Columns report $E_{ads}(Li_2S_x)$ for x = 1,4,6,8 on pristine/edge benchmarks and on S/O-bearing basal-plane defects.

Substrates	$E_{ads}(Li_2S)$	$E_{ads}(Li_2S_4)$	$E_{ads}(Li_2S_6)$	$E_{ads}(Li_2S_8)$
Zigzag-edge S[6]	0.30	0.58		0.60
S-graphene[11]		-0.76	-0.83	-0.71
OC_3	-0.627	-0.676	-0.823	-1.095
$OC_2(OH)$	-0.064	-0.152	-0.232	-0.449
$OC_2(O)$	-1.837	-1.265	-1.276	-1.737
$OC_2(O)(OH)$	-1.627	-1.032	-1.163	-1.517
OC_2-59	-1.053	-0.828	-0.981	-1.342
OC_2-59(OH)	-1.138	-1.067	-1.133	-1.589
OC_2-2OH	-1.329	-0.854	-1.036	-1.430
OC_2-2OH(OH)	-1.319	-0.837	-1.021	-1.465
pOC_2	-0.791	-0.741	-0.874	-1.256
$pOC_2(OH)$	-0.949	-0.984	-1.110	-1.464
SC_3	-0.695	-0.703	-0.838	-1.177
$SC_2(OH)$	-1.032	-1.041	-1.047	-1.465
$SC_2(O)$	-1.175	-1.078	-1.200	-1.723
$SC_2(O)(OH)$	-1.243	-1.193	-1.351	-1.774
SC_2-59	-0.770	-0.761	-0.908	-1.329
SC_2-59(OH)	-1.171	-1.139	-1.204	-1.641
SC_2-2OH	-0.904	-0.914	-0.972	-1.418
SC_2-2OH(OH)	-0.815	-1.108	-1.193	-1.305
pSC_2	-0.697	-0.742	-0.848	-1.226

References:

[1] W. Kohn, L. J. Sham, *Phys. Rev.* **1965**, *140*, A1133.
[2] G. Kresse, J. Hafner, *Phys. Rev. B* **1993**, *47*, 558.
[3] P. E. Blöchl, *Phys. Rev. B* **1994**, *50*, 17953.
[4] J. P. Perdew, K. Burke, M. Ernzerhof, *Phys. Rev. Lett.* **1996**, *77*, 3865.
[5] H. J. Monkhorst, J. D. Pack, *Phys. Rev. B* **1976**, *13*, 5188.
[6] T.-Z. Hou, X. Chen, H.-J. Peng, J.-Q. Huang, B.-Q. Li, Q. Zhang, B. Li, *Small* **2016**, *12*, 3283.
[7] Z. Zeng, W. Nong, Y. Li, C. Wang, *Advanced Science* **2021**, *8*, 2102809.
[8] Z. Lian, M. Yang, F. Jan, B. Li, *J. Phys. Chem. Lett.* **2021**, *12*, 7053.
[9] D. He, X. Zhang, L. Chen, P. Wei, W. Zhu, X. Nie, P. Zhai, Y. Huang, W. Zhao, *Advanced Materials* **2025**, *37*, 2500457.
[10] J. K. Nørskov, J. Rossmeisl, A. Logadottir, L. Lindqvist, J. R. Kitchin, T. Bligaard, H. Jónsson, *J. Phys. Chem. B* **2004**, *108*, 17886.
[11] S. P. Jand, Y. Chen, P. Kaghazchi, *Journal of Power Sources* **2016**, *308*, 166.
